# *Citrus × Clementina* Hort. Juice Enriched with Its By-Products (Peels and Leaves): Chemical Composition, In Vitro Bioactivity, and Impact of Processing

**DOI:** 10.3390/antiox9040298

**Published:** 2020-04-03

**Authors:** Mariarosaria Leporini, Monica Rosa Loizzo, Vincenzo Sicari, Teresa Maria Pellicanò, Antonella Reitano, Annabelle Dugay, Brigitte Deguin, Rosa Tundis

**Affiliations:** 1 Department of Pharmacy, Health and Nutritional Sciences, University of Calabria, 87036 Rende (CS), Italyrosa.tundis@unical.it (R.T.); 2 Department of Agricultural Science, Mediterranean University of Reggio Calabria, 89123 Reggio, Calabria, Italy; vincenzo.sicari@unirc.it (V.S.); teresa.pellicano@unirc.it (T.M.P.); 3 Department of Business and Legal Sciences, University of Calabria, 87036 Rende (CS), Italy; antonella.reitano@unical.it; 4 U.M.R. CiTCoM (CNRS, Université Paris) n°8038, Faculté de Pharmacie de Paris, Université de Paris, F-75006 Paris, France; annabelle.dugay@parisdescartes.fr (A.D.); brigitte.deguin@parisdescartes.fr (B.D.)

**Keywords:** *Citrus*, enriched juice, by-products, chemical profiles, pasteurization, antioxidants and related metabolic diseases

## Abstract

This work investigated a model for the reuse of *Citrus × clementina* Hort. by-products for the development of a functional drink able to exert antioxidant, hypoglycaemic, and hypolipidemic effects. Juice obtained from fruits collected in three different areas of Calabria (Italy) was analysed. *C. × clementina* juice from Corigliano Calabro (JF), characterized by the highest content of bioactive compounds and bioactivity, was chosen as a matrix to be enrichment with hydroalcoholic ultrasound-assisted maceration of *C. × clementina* leaf from Corigliano Calabro (CO2) and ethanol ultrasound-assisted maceration of *C. × clementina* peel from Cetraro (BC3) extracts at different concentrations. The highest phytochemical content and bioactivities were found in juice enriched with leaf and leaf + peel extracts, with particular reference to antioxidant activity. In order to estimate the effects of pasteurization, 20% (mg/100 mL) enriched juice was subjected to this process. Based on obtained data of bioactivity and sensorial analysis, *C. × clementina* by-products could be proposed as a promising source of bioactive compounds useful for the formulation of a functional drink for preventing diseases associated with oxidative stress such as type 2 diabetes and obesity.

## 1. Introduction

In a circular economy, the value of products and waste is maintained for as long as possible; they are recovered, regenerated, and reused at the end of their life [1]. This can contribute to innovation and growth in the food and beverage industry [2]. Food by-products are produced in large amounts in food industries on an annual basis worldwide. About 38% of food waste is produced during food processing. Vegetable-derived processing food waste includes peels, stems, seeds, shells, bran, and trimming residues [3]. Proper waste management plays a pivotal role in the growth of food industries [4]. The agri-food matrices containing a wide range of phytochemicals can be used as ingredients for food supplements (nutrition) or as bioactive compounds (such as polyphenols, tocopherols, dietary fiber, essential oils, unsaturated fatty acids, and peptides) to be employed in pharmaceutical and cosmetic industries [5,6,7,8,9,10,11,12]. 

*Citrus* (Rutaceae) is the most produced tree fruit crop in the world. The increase in global *Citrus* production is constant in XXI^th^ century, and annual production has reached more than 131.3 million tons [13]. 

*Citrus × clementina* Hort., a hybrid between mandarin and orange, is one of the most important crop varieties of *Citrus* in the Mediterranean area [14]. Clementine fruits grow on different continents, and Italy represents the major European producer [15]. In Calabria ( southern Italy), the cultivation of clementine is widespread due to optimal climatic conditions that have contributed to the development of food products awarded the Protected Geographical Indications (PGI) certification by the European Commission as “Clementine di Calabria” in 1997 [16]. Climatic and environmental conditions can cause variations in the chemical composition of the vegetable matrix. Indeed, the adaptation of many species to the natural environment that hosts them is a fundamental element for the assessment of biodiversity, understood as the chemical, genetic, and morphological variability of a plant species [15]. Hence, exogenous and endogenous factors can modify the presence/absence or abundance of a single component in the matrix. *C. × clementina* needs a mild climate, as constant as possible during the growing season. It is sensitive to temperature changes, especially those caused by cold winds that dry the twigs [14]. Several literature data reported the beneficial health effects of *C. × clementina* fruits and *Citrus*-derived products. Some of these properties include antioxidants, hypoglycaemic, hypolipidemic, enzymatic browning inhibition, antiproliferative, neuroprotective, and antimicrobial activities, which are related to the presence of bioactive compounds including vitamin C, carotenoids, phenolics, and essential oils [15,17,18,19,20,21,22]. 

Almost 33% of *Citrus* fruits are industrially processed for juice production; however, a large amount of *Citrus* waste including peels, segment membranes and seeds is produced. A worldwide production of 15 million tons per year of *Citrus* waste is estimated. Due to the low cost and easy availability, the residues of *Citrus* fruit, discarded as waste in the environment, should be considered a potential nutraceutical source. In fact, these by-products are rich in bioactive phytochemicals and could be recycled as value-added food supplements, which provide advantageous dietary fibre, polyphenols, and other bioactive compounds. Hence, these by-products are considered a renewable resource. The biomolecules recovered from the by-products can be used to produce functional foods and consequently offer a new opportunity for by-product reutilization. Therefore, pharmaceutical and food sectors have a common interest to obtain new natural bioactive components. The idea behind functional food is to reduce the prevalence of chronic diseases by limiting the consumption of “chemically modified” foods to give them a “healthier” appearance [23]. Functional food products have received enormous attention in the food market due to the growing interest of consumers in “healthy” foods. The Functional Foods Market was valued at 153600 million US$ in 2018 and will reach 260400 million US$ by the end of 2025, growing at a CAGR (Compound Annual Growth Rate) of 6.8% during the 2019–2025 period. 

Metabolic syndrome (MetS) is a group of risk factors, including central obesity, insulin resistance, impaired glucose tolerance, dyslipidaemia, and hypertension, that increase the risk of type 2 diabetes mellitus (T2DM), and cardiovascular disease [24]. Concerning the epidemiologic findings, the International Diabetes Federation estimated that MetS affects 25% of the population worldwide. Of that, the US population is the most highly affected, followed by Europeans [25]. In the search for both therapeutic and preventative strategies regarding MetS, the use of plants/herbs and/or their bioactive compounds are worthy of consideration [26]. The efficacy of plants and/extracts in MetS have been attributed to the diversity of active compounds with multiple mechanisms of actions that may work synergistically or potentiate the activity of each other [27,28]. 

Following our previous work in which we investigated *C. × clementina* leaf polar extracts and essential oils (EO) as sources of antioxidant and hypoglycaemic agents [15], the present study aimed to obtain a *C. × clementina* functional juice enriched with extracts of its by-products (peels and leaves) that could be used to prevent metabolic syndrome. 

For this purpose, both juice and polar extracts were investigated for their chemical profile by High-performance liquid chromatography (HPLC) analyses whereas peel essential oils were analysed by Gas Chromatography–Mass Spectrometry (GC-MS). All samples were investigated in vitro for their potential antioxidant-, hypoglycaemic-, and hypolipidemic-related effects. In particular, antioxidant properties were investigated by using four in vitro assays (ABTS, DPPH, FRAP, and β-carotene bleaching tests), hypoglycaemic effects were analysed by α-amylase and α-glucosidase inhibitory activity tests, and the hypolipidemic activity was studied by using lipase inhibitory assay. Moreover, in order to estimate the effect of pasteurization, enriched juice was subjected to this process and its impact on bioactive compounds was assessed.

## 2. Materials and Methods 

### 2.1. Chemicals and Reagents

Solvents of analytical grade were obtained from VWR International s.r.l. (Milan, Italy). 

Tween 20, ascorbic acid, Folin–Ciocalteu reagent, sodium carbonate, butylated hydroxytoluene (BHT), propyl gallate, 2,2-diphenyl-1-picrylhydrazyl (DPPH), tripyridyltriazine (TPTZ), 2,2′-azino-bis(3-ethylbenzothiazoline-6-sulfonic) acid (ABTS) solution, β-carotene, linoleic acid, Orlistat, Trizma base, 4-nitrophenyl octanoate (NPC), maltose, α-amylase from porcine pancreas, α-glucosidase from *Saccharomyces cerevisiae*, *o*-dianisidine dihydrochloride, and peroxidase/glucose oxidase (PGO) were purchased from Sigma–Aldrich S.p.a. (Milan, Italy). 

Acarbose from *Actinoplanes* sp. was obtained from Serva (Heidelberg, Germany). Caffeic acid, protocactechuic acid, *p*-coumaric acid, chlorogenic acid, vanillic acid, eriocitrin, gallic acid, apigenin, didymin, quercetin, hesperidin, neohesperidin, neoeriocitrin, naringin, narirutin, sinensetin, tangeretin, rutin, quercetin-*O*-glucoside, genistin, poncirin, luteolin, kaempferol, hesperetin, rhamnetin, umbelliferone, isopimpinellin, and bergapten were purchased from Sigma–Aldrich Chem. Co. (Milwaukee, WI, USA). Acetonitrile, formic acid, methanol, and water were HPLC-grade solvents, obtained from Carlo Erba Reagents (Milano, Italia).

### 2.2. Plant Materials 

*C. × clementina* Hort. fruits, cv “Comune”, were collected in November from healthy trees cultivated under the same climatic and culture conditions in three areas of Calabria (Southern Italy), namely, Cetraro (area D, Latitude: 39°30′59″ N, Longitude: 15°56′29″ E, 138 m above sea level), Rosarno (area E, Latitude: 38°29′13″ N, Longitude: 15°58′46″ E, 68 m above sea level), and Corigliano Calabro (area F, Latitude: 39°35’45”60 N, Longitude: 16°31’6”60 E, 210 m above sea level). The authentication was carried out by Dr. N.G. Passalacqua, Natural History Museum of Calabria and the Botanic Garden, University of Calabria (Italy).

### 2.3. Sample Preparation

*C. × clementina* fruits were washed to remove superficial contamination, dried with paper, peeled, squeezed, and the juices (JD, JE, and JF from Cetraro, Rosarno, and Corigliano Calabro, respectively) were collected in a separate container and stored at 4 °C for further analysis.

Fresh peels (0.95, 1.2 and 1.6 kg, respectively, for Cetraro, Rosarno and Corigliano Calabro) were extracted by different methodologies, such as maceration, Soxhlet apparatus, ultrasound-assisted extraction, and hydrodistillation. In particular, polar extracts were obtained by: a) Soxhlet apparatus using ethanol (1:14 g/mL, 7 cycles), b) maceration using EtOH (1:6 g/mL, 3 × 72 h) and 80% v/v hydroalcoholic solution of ethanol (1:6 g/mL, 3 × 72 h), c) ultrasound-assisted maceration using EtOH (1:7 g/mL, 3 × 1 h), and 80% v/v hydroalcoholic solution of ethanol (1:7 g/mL, 3 × 1 h). For this extraction procedure, three cycles with an ultrasonic frequency of 40 kHz at a temperature of 30 °C were conducted for each sample in a Branson model 3800-CPXH water bath (Branson, Milan, Italy). After each extraction cycle, the mixture was filtered through Whatman filter Paper 4 under vacuum, and the solvent was removed using a rotary vacuum evaporator at 30 °C. Each extraction was performed in triplicate. Site collection, sample names, extraction procedure, and yield (%) are reported in Table 1.

Essential oils were obtained by hydrodistillation of fresh peels (580, 644, and 950 g, respectively, for Cetraro, Rosarno, and Corigliano Calabro) for 3 h using a Clevenger-type apparatus. A white–yellow essential oil was obtained (3.82, 6.05, and 13.20 mL, respectively, for Cetraro, Rosarno and Corigliano Calabro). The oil was dried over anhydrous sodium sulphate, stored in hermetically sealed brown glass bottles, and kept at 4 °C before analysis. 

Site of collection, sample names and extraction procedures of juice and fresh peel extracts are reported in Table 1. This table also reported CO2 samples obtained from fresh leaves that were investigated in our previous work [15]. 

JF, characterized by a higher content of bioactive compounds and higher bioactivity (see paragraph 3.6 and 3.7), was chosen as a matrix to be enriched with its by-products for the development of a functional drink (Table 2). BC3 (ethanol ultrasound-assisted maceration of Cetraro peels) and CO2 (hydroalcoholic (80:20 v/v) ultrasound-assisted maceration of Corigliano Calabro) extracts, characterized by the highest bioactivity (see paragraph 3.6 and 3.7), were selected to be added to JF (100 mL) in diminishing proportions varying from 20% to 5% (Table 2).

In order to evaluate the impact of food processing on the phytochemical content and bioactivity of the functional juices, JFA (enriched with leaf extract), JFE (enriched with peel extract), and JFI (enriched with leaf and peel extracts), were pasteurized (Table 2) following the procedure reported by Rabie et al. [29]. The fresh juices (250 mL) were poured into dark jars inside and heated at 90 °C for 10 min using a thermostatic water bath (Branson model 3800-CPXH, Milan, Italy). Pasteurized juice was cooled to room temperature in a water bath for 30 min.

The following juices were obtained: JPFA, pasteurized enriched juice JPF with 20% (mg/100 mL) leaf extract; JPFE, pasteurized enriched juice JF with 20% (mg/100 mL) peel extract; JPFI, pasteurized enriched juice JF with 20% (mg/100 mL) peel + leaf extracts. All samples were stored at 4 °C for further analysis.

### 2.4. Quality Parameters of C. × Clementina Fruits

Twenty-five fruits for each area of growth (Cetraro, Rosarno, and Corigliano Calabro) were collected and examined for integrity and absence of insect and dust contamination. 

Physical characteristics of the fruits such as fruit weight (g), equatorial diameter (cm), longitudinal diameter (cm), fruit firmness (g/0.5 cm^2^), peel thickness (mm), total seeds per fruit, and amount of extracted juice (%) were determined. Samples were freeze-dried and stored at −20 °C until analysis. Ash, fats, crude fiber contents, total carbohydrates, and energy values were evaluated [14].

### 2.5. Quality Parameters of C. × Clementina Juice

*C. × clementina* fruits were squeezed, and the juice was centrifuged and filtered by Whatman #54 filter paper for analysis. The colour of fresh juice was measured at 25 °C using a Konica Minolta CM-700/600 d spectrophotometer (Konica Minolta Sensing, Japan). Data were expressed as higher saturation of colour or chroma (C*). The higher the chroma values, the higher colour intensity of samples is perceived by humans [30]. Total soluble solids (TSS), pH, total acidity (TA), and ascorbic acid were determined as previously reported [14]. 

The mineral elements in *C. × clementina* pulp were measured using AAnalyst 600 atomic absorption spectrophotometer with flame atomisation (Perkin Elmer, Milan, Italy). The measurements were made in hold mode with air acetylene flame. To achieve maximum sensitivity and precision, the equipment was equilibrated by alignment of the lamp and lighter and adjustment of the selected wavelength. The analytical conditions for the measurement of mineral elements were established using the respective acidified standard. The charred pulp was then ashed in a muffle furnace at 550°C until a whitish ash was obtained. The ash was treated with 5 mL of nitric acid 5 N, transferred to a volumetric flask and made up to 100 mL. All standard solutions were measured using sodium, potassium, magnesium, and a calcium hollow cathode lamp at respective wavelengths of 589, 766, 285, and 422 nm using air acetylene flame.

### 2.6. Gas Chromatography–Mass Spectrometry (GC–MS) Analyses 

Peel essential oils (BC6, BR6, BO6) were subjected to analysis by GC-MS, using a Hewlett–Packard gas chromatograph equipped with a non-polar HP-5 capillary column (30 m × 0.25 mm, 0.25 μm), associated with a Hewlett-Packard mass spectrometer (Agilent, Milan, Italy). The ionization of the sample constituents was performed under electronic impact (EI, 70 eV). The analyses were carried out with the following temperature schedule: 50 °C for 5 min, the temperature increase from 50 to 250 °C with rate 5 °C/min, and finally reach 250 °C for 10 min. Helium was used as a carrier gas. The identification of compounds was based on the comparison of the mass spectral data with the Wiley 138 library and referring to the spectral data of pure standards and compounds in the literature. Essential oils were also analysed using a Shimadzu GC17A gas chromatograph (GC) (Shimadzu, Milan, Italy), equipped with an HP-5 capillary column (30 m × 0.25 mm, 0.25 μm). Nitrogen was used as a transport gas. The conditions used are the same as those described for the GC-MS analyses.

### 2.7. Total Phenol, Flavonoid and Carotenoid Contents of Juice and Extracts

*C. × clementina* total phenol content (TPC) was evaluated by using the Folin-Ciocalteu method as previously reported [31]. The sample at a concentration of 1.5 mg/mL (0.1 mL) was mixed with a solution of Folin-Ciocalteu reagent (0.5 mL) and water (1 mL). After 1 min of incubation, 1.5 mL of 20% sodium carbonate was added, and mixture was incubated at room temperature. The absorbance was measured at 765 nm using a UV-Vis Jenway 6003 spectrophotometer (Carlo Erba, Milan, Italy). The total phenol content was expressed as mg of chlorogenic acid equivalents (CAE)/g of fresh weight (FW). Total flavonoid content (TFC) was determined spectrophotometrically using a method based on the formation of a flavonoid–aluminium complex [32]. The sample was mixed with aluminium chloride solution (2%) in a 1:1 ratio and incubated at room temperature for 15 min. The absorbance was measured at 510 nm. TFC was expressed as mg quercetin equivalents (QE)/g FW. The total carotenoid content (TCC) was determined as previously described [15]. Briefly, 1 mL of the extract was added to 0.5 mL of NaCl 5% solution, vortexed for 30 s and centrifuged at 4500 rpm for 10 min. The supernatant (100 μL) was diluted with 0.9 mL of n-hexane and measured at 460 nm. TCC was expressed as mg β-carotene equivalents/g FW.

### 2.8. HPLC–DAD Phenolic Profile

HPLC was employed in order to investigate the *C. × clementina* phenolic profile. The HPLC analysis was performed using a Knauer instrument (Asi Advanced Scientific Instruments, Berlin, Germany) and a UV-Vis diode array detector (DAD). The individual compounds were quantified by direct injection of the samples, appropriately diluted in the mobile phase and filtered through a 0.45 µm filter (Sartorius Minisart RC-4) in an HPLC system (Knauer Smartline Pump 1000), equipped with a Knauer Smartline UV detector 2600, and using a Knauer column Eurospher 100-5 C18 (150 × 4.6 mm equipped with a guard column) at 25 °C.

The solvent consisted of solution A (acetonitrile/water/phosphoric acid, 70:26:4) and solution B (potassium dihydrogen phosphate at pH 3.5). The gradient program was as follows: starting condition, 85% solution A and 15% solution B for 5 min followed by 70% solution A and 30% solution B for 20 min; successively, 50% solution A and 50% solution B for 30 min, then 25% solution A and 75% solution B for 35 min and 5% solution A and 95% solution B for 40 min, and finally 85% solution A and 15% solution B for 20 min. Analyses were performed at a flow rate of 1 mL/min, and the chromatogram was monitored at 287 nm. 

Caffeic acid, protocactechuic acid, *p*-coumaric acid, chlorogenic acid, vanillic acid, eriocitrin, gallic acid, apigenin, didymin, quercetin, hesperidin, neohesperidin, neoeriocitrin, naringin, narirutin, sinensetin, tangeretin, rutin, quercetin-*O*-glucoside, genistin, poncirin, luteolin, kaempferol, hesperetin, and rhamnetin were selected as standards. Identification of compounds was performed by comparing their retention time with those of standards and confirmed with characteristic spectra using a photodiode array detector and literature data [17]. Calibration curves, detection limits (LOD), and quantification limits (LOQ) of analytical methods for determination of phytochemicals in *C. × clementina* samples are reported in Table S1.

### 2.9. HPLC Coumarin Determination

Umbelliferone, isopimpinellin, and bergapten were selected as standards. A concentration of 10 mM was used. *C. × clementina* samples were dissolved in a MeOH/H_2_O (80:20 v/v) solution and filtered on 0.45 μM UptiDisc nylon filters (Interchim, Montluçon, France). The HPLC–DAD–UV analysis was performed on a LaChrom Elite device supplied by VWR (Fontenay-sous-Bois, France) with a D-7000 interface, an L-7200 autosampler, L-7100 pump, L-UV detector 7400, and running on EZChrom Software Elite 3.3. A Phenomenex C18 column (150 mm × 4.6 mm, 5 μm) was used for analysis and was set to room temperature. The solvent consisted of 0.1% formic acid in water (A) and 0.1% formic acid in methanol (B). Thirty μL of each sample was injected, and the chromatograms were recorded at λ = 280 nm. The gradient program was as follows: starting condition, 80% A, 20% B; 0–20 min, 80–40% A; 20–60 min, 10% A; 60–65 min, 0% A; 65–75 min, 80% A. The flow rate was set to 0.8 mL/min. The applied method showed good specificity, linearity (*r^2^* ≥ 0.9905), repeatability (RSDs < 0.02–0.04%), and intermediate precision (RSDs < 0.03–0.07%). For selected markers, this method is validated for concentrations ranging from 1.00 to 8.00 mM because recovery values are between tolerance ranges, 97–107% for umbelliferone, 84–106% for isopimpinellin, and 98–108% for bergapten, which are included in acceptable intervals (75–125%) [15]. Calibration curves, detection limits (LOD), and quantification limits (LOQ) of analytical method for determination of coumarins in *C. × clementina* samples are reported in Table S2.

### 2.10. Antioxidant Activity

The in vitro antioxidant activities of all *C. × clementina* samples were evaluated by using 2,2′-azino-bis(3-ethylbenzothiazoline-6-sulfonic) acid (ABTS), 2,2-diphenyl-1-picrylhydrazyl (DPPH), Ferric Reducing Antioxidant Power (FRAP), and β-carotene bleaching assays. 

ABTS assay was applied using the methodology previously described by Loizzo et al. [14]. A solution of ABTS radical cation was prepared by mixing 7 mM ABTS solution with 2.45 mM potassium persulphate and stored at room temperature. After 12 h, the solution was diluted with ethanol to an absorbance of 0.70 at 734 nm using a UV-Vis Jenway 6003 spectrophotometer. Dilution of extracts in ethanol were added to 2 mL of diluted ABTS^+^ solution in order to test the following concentrations from 400 to 1 μg/mL. After 6 min, the absorbance was read at 734 nm. 

DPPH radical scavenging activity was determined according to the technique reported by Loizzo et al. [14]. An aliquot of 1.5 mL of 0.25 mM DPPH radical (DPPH·) in ethanol was mixed with 12 μL of samples in order to test concentrations ranging from 1000 to 1 μg/mL. The mixture was shaken and allowed to reach a steady state at room temperature for 30 min. The bleaching of DPPH was determined at 517 nm with a UV-Vis Jenway 6003 spectrophotometer. Ascorbic acid was used as a positive control in both radical scavenging activity assays. 

In the β-carotene bleaching test, a mixture of linoleic acid, Tween 20, and β-carotene was prepared as previously described [17]. One mL of β-carotene (0.2 mg/mL in chloroform) was added to 20 μL of linoleic acid and 200 μL of 100% Tween 20. After evaporation of the solvent and dilution with water, the emulsion (288 μL) was added to a 96-well microplate containing 12 μL of samples in ethanol concentrations ranging from 100 to 2.5 μg/mL. The plate was shaken and placed at 45 °C in a water bath for 30 and 60 min. The absorbance was measured at 470 nm. Propyl gallate was used as a positive control. 

For the preparation of FRAP reagent, a mixture of 2.5 mL of 10 mM tripyridyltriazine (TPTZ) solution, 40 mM HCl, 2.5 mL of 20 mM FeCl3, and 25 mL of 0.3 M acetate buffer (pH 3.6) was prepared [15]. An aliquot of 100 μL of sample at a concentration of 2.5 mg/mL in ethanol was mixed with 2.0 mL of FRAP reagent and 900 mL of water; the absorption of the reaction mixture was measured at 595 nm after 30 min of incubation at room temperature. Ethanol solutions of known Fe (II) concentration, in the range of 50–500 μM (FeSO_4_), were used for obtaining the calibration curve. The FRAP value was expressed as μM Fe(II)/g. Butylated hydroxytoluene (BHT) was used as a positive control.

### 2.11. Relative Antioxidant Capacity Index (RACI)

RACI is an integrated statistical application used to estimate the antioxidant capacity generated by different in vitro methods [33]. Herein, data obtained from the four applied antioxidant tests were used to calculate the RACI value by using the following equation:

RACI = (x−μ)/σ

where x is the raw data, μ is the mean, and σ is the standard deviation. 

### 2.12. Carbohydrate Hydrolysing-Enzyme Inhibition Study

α-Amylase and α-glucosidase are enzymes involved in carbohydrate digestion and have been recognized as therapeutic targets for the modulation of post-prandial hyperglycaemia [34]. 

In the α-amylase inhibitory assay, the enzyme solution was prepared by adding 0.0253 g of enzyme in 100 mL of cold water, and the starch solution was prepared by stirring (at 65 °C for 15 min) 0.125 g of potato starch in 25 mL of sodium phosphate buffer (20 mM) and sodium chloride (6.7 mM) [14]. Samples were dissolved in ethanol at concentrations ranging from 1000 to 25 μg/mL, added to starch solution, and left to react with the enzyme at room temperature for 5 min. The absorbance was read at 540 nm. 

In the α-glucosidase inhibitory activity test, a maltose solution was prepared by dissolving 12 g of maltose in 300 mL of 50 mM sodium acetate buffer; α-glucosidase (EC 3.2.1.20) solution was prepared by adding 1 mg of enzyme (10 units/mg) in 10 mL of ice-cold distilled water; and O-dianisidine (DIAN) solution was prepared by dissolving 1 tablet in 25 mL of distilled water [15]. The peroxidase/glucose oxidase (PGO) system-colour reagent solution was obtained by dissolving 1 capsule in 100 mL of ice-cold distilled water. A mixture of 5 μL of sample (at concentrations ranging from 1000 to 25 μg/mL), 250 μL maltose solution, and 5 μL enzyme was left to incubate at 37 °C for 30 min. Then, 50 μL of perchloric acid was added, and the mixture was centrifuged. The supernatant was collected and mixed with 5 μL of DIAN and 300 μL of PGO and left to incubate at 37 °C for 30 min. The absorbance was read at 500 nm. Acarbose was used as a positive control in both tests.

### 2.13. Pancreatic Lipase Inhibition Assay

Pancreatic lipase inhibitory activity was determined by the 96-well plate method based on the procedure proposed by El-shiekh et al. [35]. 4-Nitrophenyl octanoate (NPC), 5 mM in dimethyl sulfoxide solution and an aqueous solution of porcine pancreatic lipase (1 mg/mL) and Tris-HCl buffer (pH 8.5) were prepared. Samples (2.5–40 mg/mL) were added to a well with 6 μL of the enzyme, 6 μL of NPC, and 279 μL of buffer. The mixture was incubated at 37 °C for 30 min. 

The absorbance was measured at 405 nm. Experiments were performed in triplicate. Orlistat was used as a positive control.

### 2.14. Sensory Analysis

Sensory evaluation was conducted by a selected and trained panel comprising 15 judges from graduate students of Science of Nutrition. 

Samples were served at 12–15 °C in tasting glasses and were coded. Each subject received 17 samples (unidentified, with randomly assigned three-digit codes): a control juice sample (without dried extracts), pasteurized (JPF), and non-pasteurized (JF), enriched juice with different extract concentrations (20, 15, 10, and 5% mg/100 mL), and pasteurized enriched juice (20% mg/100 mL). 

The evaluation was done using 9-point structured scales, 9 being the best and 1 the worst quality (colour, odour, appearance, aroma, sweetness, acidity, astringency, and mouthfeel). 

### 2.15. Statistical Analysis

All experiments were carried out in triplicate. Data are expressed as means ± standard deviation (S.D.). The concentration that yielded 50% inhibition (IC_50_) was calculated by nonlinear regression with the use of Prism GraphPad Prism version 4.0 for Windows (GraphPad Software, San Diego, CA, USA). The concentration-response curve was obtained by plotting the percentage inhibition versus concentration. Differences within and between groups were evaluated by one-way analysis of variance test (ANOVA) followed by a multicomparison Dunnett’s test (α = 0.05) that was used to compare each group with the positive control in biological assays and Tukey’s test to determine any significant difference in chemical parameters among investigated samples at different levels: * *p* < 0.1, ** *p* < 0.01, *** *p* < 0.001, **** *p* < 0.0001. Studies of the *Pearson’s* correlation coefficient (r) and linear regression, assessment of repeatability, calculation of average, and relative standard deviation were performed using Microsoft Excel 2010 software. Principal component analysis (PCA) was applied by SPSS software for Windows, version 15.0 (Chicago, IL, USA). Statistical analyses were performed using SPSS software for Windows (SPSS Inc., Elgin, IL, USA) version 22.0.

## 3. Results and Discussion

### 3.1. Quality Parameters

*C. × clementina* fruit carpometric parameters displayed some statistically significant differences (Table S3). In particular, fruits from Cetraro were characterized by a lower weight (87.19 g) and lower fruit firmness (410.19 g/0.5 cm^2^), determined on a portion of peels and ‘albedo’. Several differences were observed for equatorial and longitudinal diameters. Total soluble solids (TSS), pH, total acidity (TA), and the colour of *C. × clementina* juice were investigated (Table 3). 

The values of TA were 0.57 and 0.70 g citric acid/100 mL for JD and JF, respectively. A similar value was found for TSS. A pH ranging from 3.47 to 3.72 was measured. Significant differences were observed in the chroma value (C*), where JF presented the highest value (34.04). Similar values were found for ash, fats, protein, fiber, carbohydrates, and energy. Additionally, all samples were rich in potassium.

### 3.2. Extraction Yield and Total Phytochemical Contents

Despite the weight differences among the fruits, the difference in the percentage yield of juice was minimal, with percentages of 47.13 vs. 48.37% for JE and JF, respectively, as shown in Table 4. 

*C. × clementina* peel extraction yields (g/g) are reported in Table 4. Samples obtained by the ethanol extraction (Soxhlet) procedure showed the highest yield of extraction, i.e., 14.99, 14.42, and 14.28%, for BO1, BC1, and BR1, respectively. Different yields were observed with extracts obtained by the ultrasound-assisted maceration process with EtOH/H_2_O (80:20 v/v) as the solvent. BC2 (Cetraro) showed the highest yield followed by BO2 (Corigliano Calabro) and BR2 (Rosarno) (12.91, 11.50, and 9.48% w/w, respectively). The same trend was observed for the extracts obtained by the ultrasound-assisted maceration process with EtOH alone, with percentages of 12.48, 10.74, and 10.49% for BC5, BO5, and BR5, respectively. 

An interesting total phenol content (TPC) with a range of 29.46–31.12 mg of chlorogenic equivalents/100 mL of juice was found. Moreover, the total flavonoid content (TFC) in a range of 17.58–18.23 mg of quercetin equivalents/100 mL of juice was reported. Juice samples possessed the highest carotenoid content (TCC).

The content of bioactive substances in *C. × clementina* peel extracts was analysed (Table 4). A higher total phenol content was observed in extracts obtained by the Soxhlet extractor in which ethanol was used as solvent followed by the ultrasound-assisted maceration process in EtOH/H_2_O (80:20 v/v), compared to other extracts. In particular, the highest content was found in BC1, with values of 8.75 mg CAE/g FW, followed by BR1 with a content of 7.13 mg CAE/g FW. Interesting results were also observed for BC2 and BC3, with values of 6.30 and 6.27 CAE/g FW, respectively.

Analysis of data obtained by maceration showed that the TPC in the range of 3.45–5.34 mg CAE/g FW and the TFC in the range of 5.85–5.49 mg CAE/g FW were observed by using EtOH/H_2_O (80:20 v/v) and EtOH as solvents, respectively.

Casacchia et al. [36] reported values of 109.86 mg CAE/g FW and 61.3 mg QE/g FW, respectively, for TPC and TFC found in clementine peel extracts from fruit collected in Mirto-Crosia (Cosenza, Italy). Boudries et al. [37] evaluated the TPC in Algerian clementine peel extracts and found values ranging from 9686.2 to 11934.5 mg GAE/100g DW, with a ranking of cv Cadoux > Monreal > St Martin > Merme > Rocamora > Cheylard. 

The comparison of our data with data from the literature revealed that *C. × clementina* cv Cadoux and Merme peel extracts from Algeria were richest in TFC, with values of 1047.2 and 942.5 mg EC/100 g dried weight (DW), respectively. Additionally, Levaj et al. [38] showed a TFC of 804.26 mg/100 g DW in clementine peels collected in Algeria. 

Regarding *C. × clementina* TCC, the Soxhlet extraction procedure was the most effective to enrich extracts with this class of phytochemicals (BC1, 39.84 mg equivalent β-carotene/g FW). Cetraro samples obtained by ultrasound-assisted maceration by EtOH/H2O (80:20 v/v) and EtOH showed a TCC with values of 17.89 and 16.66 mg equivalent β-carotene/g FW, respectively, in comparison to Rosarno samples (10.42 and 10.88 mg equivalent β-carotene/g FW, respectively), and Corigliano Calabro samples (10.28 and 9.66 mg equivalent β-carotene/g FW, respectively). 

### 3.3. C. × Clementina Peel Essential Oil Profile

Constituents of *C. × clementina* peel essential oils (BC6, BR6, and BO6 from Cetraro, Rosarno, and Corigliano Calabro, respectively) are listed in Table 5. Respective yields (g/g) of 0.54% and 0.80% for BC6 and BR6 essential oils were obtained. Twenty main constituents were tentatively identified. The dominant compound was limonene, with percentages of 75.11, 85.08, and 61.31% for BC6, BR6 and BO6, respectively, followed by linalool. 

Among identified compounds, limonene is responsible for the taste of *Citrus* fruits while linalool is responsible for the flower odour [39]. Interestingly, the content of α-pinene was higher in BO6 compared to BC6, while (E)-β-ocimene was present only in BC6 (3.31%). Additionally, myrcene was present in percentages 4 times higher for BO6 compared to the other two essential oils. Different concentrations of sabinene were also observed, with values of 2.55, 0.97, and 1.52%, respectively, for BC6, BR6, and BO6. The different profile supports the hypothesis proposed by Pitarokili et al. [40] that both exogenous and endogenous factors are able to change the presence/absence or abundance of a single component within the essential oil. Limonene and linalool were the two main abundant compounds of clementine cv Oroval peel oil from Sicily [41]. 

Bermejo et al. [42] analysed the essential oil obtained from the peels of three *C. × clementina* cv, Fino, Loretina, and Marisol, from Spain. Limonene was the most abundant monoterpene followed by myrcene and linalool. This result is in agreement with Nguyen et al. [43] in which the main constituents of essential oil, obtained from Vietnam clementine peels, were limonene, myrcene, and α-pinene. Recently, Boudries et al. [20] reported, limonene, β-myrcene, and sabinene as main constituents of essential oils in peels of Algerian clementine. 

Previously, Lota et al. [44] evaluated the chemical composition of clementine essential oils of different *C. × clementina* cultivars from Corsica, namely, MA3, Nules, MA2, Hernandina, Tardia Villareal, Reina, Caffin, MacBean, Oroval, Monreal, Bruno, Tomatera, Commune, Marisol, Ragheb, and Guillermina. The content order limonene > myrcene > linalool was observed. El-hawary et al. [45] confirmed that limonene and myrcene represented the main constituents of the essential oil obtained from clementine peels from Egypt.

### 3.4. Phenolic Profile

The HPLC–DAD phenolic profile of juice showed the presence of nineteen flavonoids (Table 6). Among identified constituents, the flavanone glycoside neohesperidin was the main abundant compound with values of 80.26, 110.63, and 112.32 mg/100 mL for JE, JD and JF, respectively followed by the flavanone aglycone hesperidin with value of 40.06, 65.3, and 81.08 mg/100 mL for JE, JF and JD, respectively. Neohesperidin was 2.02 times higher in JD in comparison to JE. Significant amounts of narirutin were also detected. The flavanone-*O*-glycoside didymin was present in concentration of 3.85, 4.17, and 5.51, respectively, for JD, JE, and JF. Chlorogenic acid, vanillic acid, caffeic acid, and gallic acid were also quantified. 

A similar trend was observed by Rapisarda et al. [46] for fruits collected in Acireale, Sicily. In the same study, the juice of hybrid Omo-narirutin was not the second most abundant flavanone glycoside after hesperidin. Higher values were reported by Milella et al. [47], who found a hesperidin content ranging from 63.98 to 165.88 mg/L for Etna hybrid and Rubino cultivars, respectively. 

Previously, Xu et al. [48] quantified narirutin, hesperidin, naringin, and neohesperidin in different *Citrus* varieties (Wase-Satsuma, Satsuma, Ponkan, Bendizao, Manju, new variety hybrid 439, and Zhuhong). Interestingly, the flavanone glycoside neohesperidin represented the main abundant compound in *Citrus* juice from China in our data but was not detected together with naringin. On the contrary, Nogata et al. [49] reported the presence of rutin in significant amounts in *C. × clementina* juice. 

The HPLC–DAD phenolic profile of *C. × clementina* peel extracts is reported in Table 7. Apigenin, caffeic acid, eriocitrin, neoeritrocin, quercetin-3-*O*-glucoside, quercetin, hesperidin, poncirin, luteolin, sinensetin, and tangeretin were selected as markers and were quantified. Hesperidin was the dominant compound with concentrations in the range of 100.26–1093.36 mg/100 g FW. In addition, notable quantities of sinensetin (19.56–37.09 mg/100 g FW), tangertin (5.43–8.31 mg/100 g FW), and luteolin (3.02–8.36 mg/100 g FW) were observed. Interestingly, neoeritrocin was absent in samples collected in Rosarno and Corigliano Calabro, but present in the range of 0.68–4.89 mg/100g FW in Cetraro samples. Eriocitrin was also absent in Rosarno extracts, but present in low concentrations in BO3 and BO5 and in a range of 1.56–3.75 mg/100 g FW in Cetraro samples. High variability was also found for caffeic acid. This compound was present in concentrations of 10.87 and 8.99 mg/100g FW in BR5 and BR4, respectively, but absent in Corigliano Calabro extracts (BO4 and BO5) obtained with the same extraction technique. Quercetin-3-*O*-glucoside and quercetin were absent in BR4 and BR5 samples, while notable contents were reported for BC1 (11.26 and 8.89 mg/100g FW, respectively, for quercetin-3-O-glucoside and quercetin).

Bermejo et al. [42] reported the phenolic profile of *C. × clementina* peels cv Fino, Loretina, and Marisol collected in Spain. Hesperidin was found in concentrations ranging from 33.5 to 38.64 mg/g DW, whereas sinensetin was in the range 0.15–0.25 mg/g of DW. Values of 0.27, 0.69, and 0.37 mg/g DW were found for tangeretin in cv Fino, Loretina, and Marisol, respectively. A lower content of hesperidin (47.22 mg/100 g DW) was found in clementine peel extract from Corsica [38]. The high amount of hesperidin in clementine peels was confirmed by Tumbas et al. [50], with values in the range 0.39–15.3 mg/g DW. These results are in agreement with our data and with those reported by Nogata et al. [40] reported that the most abundant constituent of clementina peels was hesperidin (1800 mg/100 g FW) followed by narirutin (57.8 mg/100g FW) and diosmin (35.4 mg/100g FW); the latter was not selected by us as a marker. 

### 3.5. Coumarin Determination

In order to exclude the presence in *C. × clementina* samples of the most common coumarins (umbelliferone, isopimpinellin, and bergapten), HPLC–DAD analysis was performed. 

Furanocoumarins have controversial effects on humans, acting as potential photosensitizers and interacting with drugs with inhibition of the intestinal cytochrome P450-3A4 [51,52]. For patients undergoing drug therapy, the inhibition of cytochrome P450-3A4 by furanocoumarins may lead to a higher concentration of drug in the blood, which in turn can cause serious side effects such as heart rhythm disturbances or respiratory depression [53]. Removing furanocoumarins from food implies additional costs and might alter the product quality [54]. 

The obtained data excluded the presence of coumarins in all investigated samples. Considering that the content of coumarins is strictly regulated in foods, the absence of these phytochemicals in our bioactive samples represented an additional value for their potential industrial application.

Dugrand-Judek et al. [55] reported that environmental factors, such as exposure to air and water pollution, stimulated furanocoumarin biosynthesis. Additionally, the presence of these secondary metabolites is influenced by phenotypic diversity and the intraspecific chemo-diversity of *Citrus* species and suggests that plants related to *C. maxima*, *C. micrantha*, *C. lemon*, and *C. hystrix* accumulated these compounds in high amounts. *C. deliciosa* and related species appeared almost devoid of them. Concerning hybrids, their corresponding chemotypes appeared to be inherited from respective ancestral taxa, with a prevalence of *C. maxima*, *C. lemon*, and *C. hystrix* related species and hybrids. More recently, Ramírez-Pelayo et al. [56] investigated the presence of coumarins and furanocoumarins in the peel extracts from *C. sinensis* var. Valencia, *C. reticulata* var. Arrayana and Oneco, *C. aurantifolia* var. Pajarito, *C. × limonia,* and *C. latifolia*. The coumarin profile is dependent on the species. Bourgaud et al. [57] reported a content of isopimpinellin and bergapten in clementine peel extract of 1.40 and 0.96 mg/kg of FW, respectively. Bergamottin was found in trace amounts [58].

### 3.6. Antioxidant Activity

An antioxidant is defined as a molecule capable of reducing or inhibiting the oxidation of other molecules, whereas a biological antioxidant is defined as “any substance that when present at low concentrations compared to those of an oxidable substrate significantly delays or prevents oxidation of the substrate”. Recently, polyphenols/flavonoids found in plants have attracted much attention among researchers as a new natural antioxidant. Several methods were developed for measuring the total antioxidant capacity of a matrix; these assays differ in their chemistry such as the generation of different radicals and/or target molecules. 

Different antioxidant compounds may act in vivo through different mechanisms; no single method can fully evaluate the antioxidant activity of a matrix. For this reason, in this work, the antioxidant properties of samples were investigated using different methods: 2,2′-azinobis (3-ethylbenzothiazoline-6-sulfonic acid) (ABTS), 1,1-diphenyl-2-picrylhydrazyl (DPPH), FRAP, and β-carotene bleaching tests. The radical scavenging activity of samples was examined using the DPPH radical and ABTS radical cation. ABTS^+^ and DPPH^.^ radicals have a different stereochemistry and a different training mechanism and therefore, after reaction with antioxidants, they show a qualitatively different response to the inactivation of their radical [14]. 

The potential of *C. × clementina* samples to inhibit lipid peroxidation was assessed using the β-carotene bleaching test. The ability of sample to induce the reduction of TPTZ-Fe^3+^ was measured with the FRAP test. 

A concentration-dependent activity was observed for all samples independently by the applied methods (Table 8). Juice obtained from fruits collected in Corigliano Calabro exhibited the highest radical potential, with IC_50_ values of 81.13 and 27.82 μg/mL for DPPH and ABTS tests, respectively, followed by the JD (IC_50_ of 82.43 and 33.63 μg/mL for DPPH and ABTS, respectively). Moreover, in the β-carotene bleaching test, JF presented the highest protection of lipid peroxidation with percentages of 31.33 and 34.20%, respectively, after 30 and 60 min of incubation. In the FRAP test, the activity of the juices was minimal; JF showed an IC_50_ value of 5.70 μM Fe (II)/g.

The *Pearson’s* correlation coefficient is reported in Table S4. In particular, the most significant correlations were found between the content of narirutin and DPPH, with *r* = 0.87. A positive correlation between naringinin, eriocitrin, and *p*-coumaric acid was found in the β-carotene bleaching test. In addition, the *Pearson’s* correlation coefficient was positive between caffeic acid, protocatechuic acid, and tangeretin and FRAP, with *r* = 0.80, 0.81, and 0.79, respectively. However, *Citrus* is a complex matrix, and for this reason, the contribution to the bioactivity of minor compounds should not excluded [59]. 

The RACI value of each juice was calculated as the mean of standard scores transformed from the raw data generated with different antioxidant methods. The difference in units and variances in the raw data had no influence on the RACI. Stepwise regression between the RACI and different chemical methods revealed that (a) each of the assays was selected as a significant variable with no single applied method being removed, (b) each method contributed the same weight in building RACI, and (c) the regression was highly significant (*r* = 1, *p* < 0.001). Therefore, the RACI of each juice is a scientific combination of data from different antioxidant methods with no unit limitation and no variance among methods which allows easier comparison of antioxidant data. Based on the RACI data, the following antioxidant rank order was found: JF > JD > JE (Table 8). This trend clearly showed that JF had the highest antioxidant potential. 

More recently, Casacchia et al. [36] reported the antioxidant potential of *C. clementina* Hort. ex. Tanaka pulp with an IC_50_ value of 100.3 mg/mL. Only hesperidin, naringin, and naringenin contents were investigated (86.8, 87.69, and 107 ppm, respectively). *C. clementina* juices obtained from fruits collected from flood plains, hills and coastal plains of Sibari (Italy) were investigated by Loizzo et al. [14]. The authors demonstrated that area of fruit collection positively influenced the bioactivity of the juice. Indeed, juice obtained from fruits collected on the hill was characterized by a higher content of bioactive compounds (neohesperidin, hesperidin, and narirutin) and antioxidant activity. These data are in accordance with our results. 

Boudries et al. [35] reported the strong radical scavenging potential of clementine pulp with IC_50_ values from 1.14 to 1.91 mg/mL for Merme and St Martin cultivars, respectively. No significant differences in radical scavenging activity were found for Safor, Fortune, Kara, and Murcott juice, while Garbí juice showed the lowest value of DPPH activity. This value can be related to the lower level of vitamin C in Garbí mandarins (21.19 mg/100 mL) [18]. A similar phenolic profile was found for our juices, except for neoeritrocin, naringenin, and didymin. Indeed, JD, JE, and JF presented a higher content of these compounds. 

Previously, Xu et al. [37] analysed the antioxidant activity of mandarin and sweet orange. A percentage inhibition of the DPPH radical from 23.69 to 61.62% for Manju and hybrid 439, respectively, was found. In all investigated juices, naringin and neohesperidin were not detected. Russo et al. [22] confirmed the radical scavenging potential of clementine mandarins (Caffin, Fedele, Ragheb, and RA89) in ABTS test, with values from 23.77 to 25.52 mg Trolox equivalent/100 mL of juice. The phytochemical content of *Poncirus trifoliata* juice and its antioxidant effects were reported by Tundis et al. [17]. The juice had IC_50_ values of 30.38 and 86.77 mg/mL, respectively, for DPPH and β-carotene bleaching tests. Similar phytochemicals were investigated, but hesperidin, naringin, and chlorogenic acid were the most abundant compounds in this juice (129.33, 115.79, and 112.54 mg/mL, respectively). Loizzo et al. [60] analysed *Citrus limon* cv Femminello comune juice, an Italian IGP (Protected Geographical Indication) product, for antioxidant potential. IC_50_ values of 40.3, and 46.5 mg/mL, respectively, for DPPH and ABTS tests, and 49.7 mM Fe(II)/g for the FRAP test were found. A comparison between lemon juice and our *C. × clementina* juice revealed a lower content of hesperidin and neohesperidin, while eriocitrin was found in higher concentration (16.7 mg/100 mL). 

The antioxidant activity of *C. × clementina* peel extracts is shown in Table 9. All tested samples showed antioxidant activity in a concentration-dependent manner. 

The greatest antioxidant potential was found for Cetraro samples in the DPPH test. In particular, BC3 showed an IC_50_ value of 45.79 μg/mL followed by BC2 (IC_50_ value of 52.58 μg/mL). Interesting results were also obtained for BR1, with an IC_50_ value of 68.13 μg/mL.

In the ABTS test, both BC2 and BO5 showed the lowest IC_50_ value of 8.22 μg/mL, followed by BR3 (IC_50_ value of 9.47 μg/mL). All samples obtained by the Soxhlet extractor using EtOH as a solvent exhibited a greater ability than other extracts to inhibit lipid peroxidation after 30 min of incubation. While, after 60 min of incubation, the best results could be attributed to samples collected in Rosarno and Cetraro and obtained by the ultrasound-assisted maceration process in EtOH (IC_50_ values of 8.78 and 16.47 μg/mL, respectively). A positive correlation was found between caffeic acid and the β-carotene bleaching test after 30 min incubation (*r* = 0.78). 

In the FRAP test, BR3 showed the greatest ability to reduce iron ions (54.95 μM Fe (II)/mg), followed by BR4 and BC3 (40.46 and 34.28 μM Fe (II)/mg, respectively). 

Concerning essential oil activity, several differences were displayed in the β-carotene bleaching test after 30 min of incubation. In fact, both BR6 (Rosarno) and BO6 (Corigliano Calabro) were more active than CE6 (Cetraro). The same observation was observed in the FRAP test in which BO6 and BR6 presented higher activity. No differences were recorded in radical scavenging activity data evaluated by DPPH and ABTS tests. Limonene, the main abundant compound in *C. × clementina* essential oils, was positively correlated with the ABTS test (*r* = 0.99). Sabinene, (E)-β-ocimene, β-sinensal, and α-sinensal presented a positive correlation with the β-carotene bleaching test (t = 30 min), with *r* values of 1.00, 1.00, 0.99, and 0.98, respectively. The same compounds positively correlated with the β-carotene bleaching test (t = 60 min), with *r* values of 1.00, 0.99, 1.00, and 1.00, respectively.

RACI was used to extrapolate samples with the highest antioxidant potential. Generally, extracts obtained by clementine fruits collected in Cetraro and Rosarno presented the highest antioxidant potential. In particular, as shown in Table 9, samples BC3 and BC1 are noteworthy. A promising antioxidant activity was found also for BR3 and BR1.

Casacchia et al. [36] reported the antioxidant activity of *C. clementina* Hort. ex. Tanaka peel extract with an IC_50_ value of 96.7 μg/mL in the DPPH test. In this extract, only hesperidin, naringin, and naringenin were investigated (193.3, 63.69, 89 ppm, respectively). Previously, Ghasemi et al. [61] reported the DPPH radical scavenging potential of *C. × clementina* peel in methanol extract with an IC_50_ value of 1.7 mg/m, while in the study conducted by Levaj et al. [38], a value of 32.2 mmol Fe^2 +^/100 g of FW was found in the FRAP test. Only hesperidin, narirutin, and naringin were quantified in this peel extract. The aqueous extract obtained from the peels and analysed by Kang et al. [62] showed lower antioxidant potential, with a 43.0% inhibition in the DPPH test. 

Previously, Loizzo et al. [63] investigated the antioxidant potential of *C. aurantifolia* peels in methanol and *n*-hexane extracts. The methanol extract had a DPPH radical scavenging activity with an IC_50_ value of 78.3 μg/mL. Promising results were obtained for protection of lipid peroxidation by using the same extract (IC_50_ values of 25.5 and 36.4 μg/mL for 30 and 60 min incubation, respectively). Values of 18.7 Trolox equivalent antioxidant capacity (TEAC) for ABTS and 146.0 μM Fe(II)/g for FRAP were observed. The results obtained for the *n*-hexane extract had an IC_50_ of 131.1 μg/mL for DPPH and 9.7 and 18.5 μg/mL for the β-carotene bleaching test after 30 and 60 min of incubation, and values of 36.2 TEAC for ABTS and 171.6 μM Fe(II)/g for FRAP. The HPLC flavonoid profile of methanol extracts of *C. aurantifolia* peels revealed the presence of rutin, apigenin, quercetin, kaempferol, and nobiletin, but hesperidin and tangeretin were not detected.

### 3.7. Effect on Carbohydrate-Hydrolysing Enzymes and Lipase 

Several research articles show that oxidative stress, obesity, and T2DM are strictly related [64]. For this reason, *C. × clementina* samples were tested for potential inhibitory activity against enzymes linked to the metabolic syndrome. 

The inhibition of carbohydrate-hydrolysing enzymes α-amylase and α-glucosidase was investigated, and results are reported in Table 10. Juice samples inhibited both enzymes in a concentration-dependent manner. Generally, the most promising activity was found against α-glucosidase. In particular, JF exhibited the highest inhibitory activity with an IC_50_ value of 67.19 μg/mL, followed by JE with an IC_50_ value of 77.79 μg/mL (*p* < 0.0001, α = 0.05). However, several differences were observed against α-amylase. In fact, JF (IC_50_ of 139.89 μg/mL) was 1.3 times more active than JE (IC_50_ of 243.24 μg/mL). 

*Pearson’s* correlation coefficient revealed a positive correlation between chlorogenic acid and α-glucosidase, with an *r* value of 0.69, while TPC was positively correlated with α-amylase. 

Pancreatic lipase (PL) inhibition is one of the most largely studied mechanisms to combat obesity. The inhibition of this enzyme delays the digestion of triglycerides to absorbable free fatty acids with reduction of postprandial hypertriacylglycerolemia. All juice samples were able to inhibit the PL enzyme in a concentration-dependent manner as shown in Table 10. A promising anti-obesity potential was found for JF with an IC_50_ value of 179.32 μg/mL followed by JD with an IC_50_ value of 192.14 μg/mL. A similar value was observed for JE (IC_50_ value of 197.69 μg/mL). 

Several *Citrus* juices are able to exert hypoglycaemic effects. The inhibition of carbohydrate-hydrolysing enzymes α-amylase and α-glucosidase of *C. clementina* juice was investigated by Loizzo et al. [14]. In particular, juice from hills exhibited the highest inhibitory activity, with an IC_50_ value of 77.79 μg/mL, followed by coastal plain juice. No significant differences were observed against α-amylase, with IC_50_ values ranging from 226.69 to 243.24 μg/mL for hill and coastal plain juice. More recently, Loizzo et al. [60] reported promising α-amylase and α-glucosidase inhibitory activities of *C. limon* juice, with IC_50_ values of 40.3 and 46.5 μg/mL, respectively. The hypoglycaemic effects of *P. trifoliata* juice were analysed via inhibition of carbohydrate-hydrolysing enzymes by Tundis et al. [17]. Juice inhibited α-amylase and α-glucosidase enzymes with respective IC_50_ values of 138.14 and 81.27 μg/mL. The administration of *C. paradisi* juice was found to significantly reduce rapid blood glucose levels without any effect on 1.5-h plasma insulin levels [65]. Mollace et al. [66] demonstrated that bergamot juice extract, administered for 30 days to Wistar rats and 237 patients affected with hyperlipidaemia associated or not associated with hyperglycaemia was able to induce a significant decrease in blood glucose level in both rats and patients. The polyphenol fraction of this extract was characterized by neoeriocitrin (7.7%), naringin (6.3%), and neohesperidin (7.2%). Recently, Casacchia et al. [36] reported the effect of hybrid Tacle^®^ (TC), a crossbreeding of *C. × clementina* and Tarocco tetraploids. Results suggested that the TC-edible portion extract was able to influence anthropometric values and lipid and glucose metabolism in a rat model of obesity and metabolic syndrome and for this reason could be included in dietary supplements for the management of metabolic disorders.

All investigated peel extracts showed inhibitory activity in a concentration-dependent manner against α-amylase and α-glucosidase (Table 10). The Cetraro extract obtained by the ultrasound-assisted maceration process with EtOH (BC3) had the highest inhibitory activity, with IC_50_ values of 71.79 and 79.73 μg/mL against α-glucosidase and α-amylase, respectively. Interesting results were also observed against α-glucosidase for BC4 (IC_50_ value of 101.91 μg/mL) and for BR4 against α-amylase (IC_50_ of 128.5 μg/mL). BR4 was characterized by a considerable tangeretin content, probably responsible of the enzyme inhibitory effect. 

Among *Citrus* phytochemicals, flavonoids are mainly involved in the management of T2DM. These compounds are able to (i) inhibit carbohydrate-hydrolysing enzymes [17]; (ii) inhibit sodium-dependent glucose transporter 1 (SGLT1) [67] (iii) stimulate insulin secretion; (iv) reduce hepatic glucose output; and (v) enhance insulin-dependent glucose uptake [68]. In particular, the main abundant flavonoid of *C. × clementina*, neohesperidin, inhibited both α-amylase and α-glucosidase in a concentration-dependent manner and was more active than acarbose. Additionally, Jia et al. [68] demonstrated that neohesperidin reduced serum glucose and glycosylated serum protein *in vivo*. Other bioactive compounds are didymin, which had an IC_50_ value of 4.20 μM against α-glucosidase, followed by naringin (IC_50_ value of 10.33 μM), narirutin (IC_50_ value of 14.30 μM), and hesperidin (IC_50_ value of 15.89 μM). This last flavanone glycoside was able to inhibit α-amylase with an IC_50_ value of 26.04 μM. The most active against α-amylase was neoeriocitrin with an IC_50_ value of 4.69 μM [17]. Previously, Shen et al. [69] studied the effect of hesperidin, naringin, neohesperidin, and nobiletin on amylase-catalysed starch digestion, pancreatic α-amylase and α-glucosidase, and glucose utilization. All investigated flavonoids were able to inhibit amylase-catalysed starch digestion. Neohesperidin and naringin principally inhibited amylose digestion, whereas hesperidin inhibited both amylose and amylopectin digestion. This demonstrated that this flavonoid could prevent the progression of hyperglycaemia in T2DM patients by a complex mechanism that involves the binding of starch, an increase of glycolysis and glycogen concentration, a lower level of gluconeogenesis, elevated oral glucose tolerance and insulin sensitivity, and decreased insulin resistance. Moreover, the hydrolysis of starch by amylase is inhibited by vitamin C alone and the vitamin C–Cu complex, the latter exerting greater inhibition [14].

Generally, extracts obtained by the ultrasound-assisted maceration procedure in both EtOH and EtOH/H_2_O showed a higher activity against lipase compared to other extracts. Promising anti-obesity potential was also found for BC3, which had an IC_50_ value of 112.0.6 μg/mL against lipase followed by BO3 (IC_50_ value of 132.37 μg/mL). 

Among *Citrus* flavonoids, Zeng et al. [70] demonstrated that narirutin and didymin were able to inhibit the lipase enzyme with IC_50_ values of 58.98 and 67.30 μg/mL, respectively. Previously, Bustanji et al. [71] reported the ability of chlorogenic, caffeic, and gallic acids to inhibit the LP and hormone-sensitive lipase (HSL) activities in a dose-dependent manner, but with different potencies. In fact, gallic acid was found to be the most potent (IC_50_ 10.1 and 14.5 μg/mL for PL and HSL, respectively) followed by caffeic acid (IC_50_ 32.6 and 40.1 μg/mL for PL and HSL, respectively). The most promising activity for chlorogenic acid was found against HSL with activity 4.5 times higher in comparison to PL. More recently, Buchholz & Melzig [72] reported the PL inhibitory activity of hesperidin and neohesperidin and demonstrated that the replacement of rutinose of hesperidin by neohesperidose caused a decrease in the inhibitory activity against PL. Moreover, hydroxy function in position 3′, and methoxy function in position 4′, favoured inhibition. Kamel et al. [73] suggested the potential benefits of the water extract of *C. reticulata* peel for obesity and fat reduction in adolescents. Clinical trials were done in a double-blind, placebo- controlled study and included 40 obese adolescent subjects. Group A participants received an 800 mg of dry extract daily, and group B received a placebo; both groups received three meals (2000 kcal/day) throughout the study. The isolated flavonoid molecules from *C. reticulata* were hesperidin, naringin, acacetin, rutin, and quercetin, found in higher amounts. The results showed a significant reduction in BMI and waist circumference after 4 and 8 weeks of supplementation of *C. reticulata* when compared with the placebo group. Additionally, results suggested the *C. reticulata* peel extract is well-tolerated and is an effective ingredient for weight management. Dallas et al. [74] investigated the efficacy and safety effects of Sinetrol-XPur (polyphenolic *Citrus* dry extract) in weight management, metabolic parameters, and glycaemic and oxidative status. Sinetrol-XPur is a proprietary polyphenolic-rich fruit extract (red orange, grapefruit, sweet orange, and guarana). It was standardized to contain at least 90% of total polyphenols (expressed as catechin), at least 20% of total flavanones (expressed as naringin), and between 1% and 3% of natural caffeine. In a 12-week, randomized, double-blind, placebo-controlled trial, Sinetrol–XPur was given to overweight subjects twice daily with meals in the tested group *versus* a placebo group. Subjects were instructed to take one capsule at breakfast and one capsule at lunch for a total of two capsules per day or 900 mg. Waist and hip circumference and abdominal fat were decreased in the Sinetrol-XPur group as compared with the placebo group. Oxidative stress was lowered as seen by the respective reduction of and the increase in superoxide dismutase and glutathione. No adverse effects were observed.

### 3.8. PCA 

In the present study, PCA was performed to group and separate the variables analysed in the *C. × clementina* juice, obtained from fruits collected in different areas. Results revealed that the first two principal components explained total variance completely, i.e., 100%. The loadings of the first and second principal components (PC1 and PC2) accounted for 51.45 and 48.55% of the variance, respectively (Figure S1). The first component (PC1) was highly positively correlated with TCC, vitamin C, chlorogenic acid, gallic acid, hesperidin, narirutin, neohesperidin, quercetin, tangeretin, caffeic acid, protocateic acid, *p*-coumaric acid, FRAP, yields, ash, fiber, carbohydrates, pH, potassium, and calcium. PC2 was positively correlated with TPC, TFC, didymin, neoeriocitrin, nobiletin, poncirin, sinensitin, FRAP, β-carotene bleaching for 30 min, β-carotene bleaching for 60 min C* peel, C* pulp, phosphorus, and magnesium. α-Glucosidase, lipase, and α-amylase inhibitory activity, °Brix value, and radical scavenging activity were negatively correlated withPC1 and PC2. 

The scores plot was used to gain an overview of the similarities or differences among the juices. The analysis demonstrated that among the juices analysed, JF was located in the top right quadrant, which represents the highest FRAP, vitamin C, tangeretin, p-coumaric acid, caffeic acid, gallic acid, protocatechuic acid, poncirin, apigenin, sinensitin, didymin, naringin, quercetin, potassium, and magnesium. The juice JD was located in the lower right quadrant, which represents hesperidin, fiber, narirutin, and TCC. The JE juice was located in the lower left quadrant. Among these, JE had the best features with reference to the variables α-amylase, α-glucosidase, and lipase. 

PCA confirmed that JF had the highest bioactive capacity. Thus, the present results provided the basic data for choosing juice with higher antioxidant activity for direct consumption or for production of a functional drink. 

For extracts, PCA applied to the data explained 71% of the total variance (Figure S2). The first component (PC1) explained 46% of all information and separated the extracts, which had the highest levels of poncirin, quercetin, quercetin-3-O-glucoside, and eriocitrin (BC4 and BC5). The second component (PC2) that discriminated BC1, BC2 and BC3 by the levels TPC, TFC, TCC, hesperedin, neoeriocitrin, sinensitin, tangeretin, and lipase explained 25% of the total variance. It is noteworthy that Cetraro extracts were separated by the other extracts probably due the high content of bioactive compounds.

### 3.9. Enriched Juice 

#### 3.9.1. Quality Characteristics

The *C. × clementina-*enriched juice parameters evaluated in this study including TSS, pH, acidity, and colour were investigated (Table S5). The enrichment with *Citrus* by-products did not result in significant changes compared to control juice. Minimal variations were observed in the colorimetric measurement. The enrichment with peel extracts showed similar values to the control juice, while the enrichment with leaf extract made the juice less bright. The total phenols, flavonoids, and carotenoid content was evaluated in all enriched juices (Table S5). As expected, enrichment with *Citrus* by-products resulted in an increase in the total phytochemical content compared to the control juice (JF). Furthermore, results showed that JFA had higher TPC, by 10%, compared to JF. A lower value of about 8% was found with JFE. 

JFB and JFA exhibited a TFC content of 26.11 and 28.44 mg QE/100 mL, respectively. In addition, JFA had an increase of 10% in the TFC compared with the control (JF). In JFI, an increase of 8% was observed. As observed for TPC, the enrichment with JFE showed a lower increase in the TFC value, about 6%, compared to control juice. No significant differences were recorded in TCC for JFE, JFL, and JFL, with values of 62.45, 62.15, and 61.98 mg equivalents of β-carotene/100 mL, respectively. Furthermore, results showed that JFE and JFF increased TCC by 8 and 7%, respectively, compared to control juice. In JFA, an increase of only 7% compared to JF was observed.

#### 3.9.2. Antioxidant Activity 

Table 11 shows the antioxidant activity of enriched juices. Generally, the increase in antioxidant activity was significantly higher in juices enriched with leaf extracts at a concentration of 20% (mg/100) except for the ABTS test in which JFI showed the highest activity.

In the DPPH test, JFA showed an increased radical scavenging potential of 28%, compared with JF control. Additionally, a promising result was observed also with JFB, which increased the antioxidant activity of the juice by about 24%, followed by JFE (13%). 

In the ABTS test, higher radical scavenging activity was observed for JFI followed by JFA. A similar value was found for JFE. JFI showed an increase of 22% in antioxidant activity as compared to the juice control. In JFA and JFE, an increase of 19 and 15%, respectively, was observed. 

The potential of *C. × clementina* juice and enriched juices to inhibit lipid peroxidation was assessed using the β-carotene bleaching test. Generally, enrichment increases protection against lipid peroxidation. JFA showed an increase of 21 and 30% in β-carotene bleaching test, respectively, after 30 and 60 min of incubation, compared to JF. In JFI, an increase in lipid peroxidation protection of 18 and 27%, respectively, after 30 and 60 min of incubation, was observed. A similar result was found for JFE, i.e., 18 and 25%, respectively, after 30 and 60 min of incubation, compared to JF. 

In the FRAP test, the antioxidant activity in enriched juice decreased as follow: JFA > JFI > JFB, with values of 100.43, 99.28, and 98.36 μg Fe(II)/g, respectively. Among them, JFA had an increase of 28% in antioxidant activity compared to JF. 

*Pearson’s* correlation coefficient was calculated. A positive correlation between TPC, TFC, TCC, and antioxidant activity was found. In particular, *r* values of 0.88 and 0.97 were found between TPC and DPPH and the β-carotene bleaching test after 30 min of incubation. Moreover, TFC was positively correlated with the DPPH and FRAP test, with *r* values of 0.93 and 0.92, respectively. Regarding TCC, a positive correlation was found only with the β-carotene bleaching test after 60 min of incubation (*r* = 0.93). 

#### 3.9.3. Effect of Enriched Juice on α-Amylase and α-Glucosidase and Lipase 

The juice enriched with leaf extract at a concentration of 20% (mg/100 mL) showed an increase of 25% in α-amylase inhibitory activity followed by JFI (22%) as compared to the juice control (Table 12).

*Pearson’s* correlation was calculated. In particular, higher positive correlations were found between TFC and α-amylase, α-glucosidase and lipase, with *r* = 0.98 and 0.97, and 0.94, respectively. Positive correlations were also observed between TPC and α-amylase (*r* = 0.91) and α-glucosidase (*r* = 0.91). Similarly, TPC and TCC were positively correlated with lipase, with *r* = 0.87, and 0.84, respectively.

#### 3.9.4. Sensory Analysis

The sensory assessment is one of the main aspects for designing new foods because changes in aroma, texture or colour of the original matrices can significantly affect the consumer’s acceptance. The evaluation was done using a 9-point structured scale, with 9 being the best and 1 the worst product quality (Table 13). Colour is the first sensorial quality that attracts attention and influences the consumer’s choice. Both colour and the appearance of peel-enriched juice were more attractive than those of the natural juice. The same result was observed for odour, and in fact, JFD obtained the highest score. Regarding aroma, testers found no significant difference between peel-enriched juice and the control. The control juice was the most acceptable for sweetness, astringency, and mouthfeel. The addition of the extracts did not cause a reduction in product acceptance in relation to the acidity.

### 3.10. Pasteurization Process 

Thermal processing is major processing technology in the food industry. The purpose of thermal processing is to extend the shelf life of food products without compromising food safety. Pasteurization is the process that uses relatively mild heat treatment to kill key pathogens and inactivate vegetative bacteria and enzymes to make food safe for consumption. Pasteurization is based on time-temperature combination processes applied to food products to achieve intended target lethality. We decided to pasteurize JF as the juice control and the enriched juices (JFA, JFE, and JFI) to evaluate the impact of the process on the phytochemical content and bioactivity of the obtained functional products. 

#### 3.10.1. Quality Parameters

The *C. × clementina* pasteurized, enriched juice (JPFA: pasteurized, enriched juice JPF with 20% leaf extract; JPFE: pasteurized, enriched juice JF with 20% peel extract; JPFI: pasteurized, enriched juice JF with 20% peel +leaf extracts 1:1) were investigated for their quality parameters such as TSS, pH, acidity, and colour analysis (Table S6) and were compared to juice control (JPF). The process did not result in significant changes compared to untreated juice. Minimal reductions (5%) were observed in the colorimetric measurement. 

The TPC, TFC, and TCC after pasteurization process were evaluated (Figure S3). Results showed an increase in phytochemicals in all samples. The total phenol content decreased as follows: JPFA > JPFI > JPFE, with values of 28.06, 24.01, and 24.34 mg CAE/100 mL, respectively. Furthermore, the results showed that in JPFA, an increase in the TPC by 12% compared to JPF, was observed. JFI showed an increase of 8%. The same value was observed for JFE. 

The TFC in pasteurized, enriched juice decreased as follow: JPFA > JPFI > JPFE, with values of 14.51, 12.55, and 11.83 mg QE/100 mL, respectively. JPFA showed an increase of 6% in the TFC compared with JPF. In JFI and JFE, an increase of 4%, was observed. 

The TCC in pasteurized, enriched juice following the trend JFE > JFI > JFA with values of 42.41, 37.22, and 31.23 mg equivalents of β-carotene/100 mL, respectively. In particular, JPFE increased the TCC by 28% compared to JPF. A similar situation was observed with JPFI (22%). 

#### 3.10.2. Antioxidant Activity 

**Figure 1:**
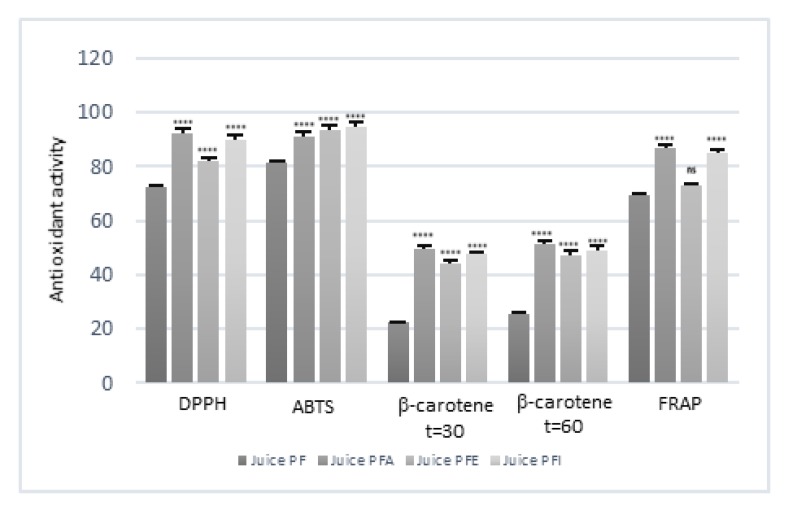
Antioxidant activity of *C. × clementina* pasteurized enriched juice. JPF: pasteurized juice JF; JPFA: pasteurized, enriched juice JPF with 20% leaf extract; JPFE: pasteurized, enriched juice JF with 20% peel extract; JPFI: pasteurized, enriched juice JF with 20% peel + leaf extracts 1:1. Data are expressed as means ± S.D. (*n* = 3). DPPH, ABTS, and β-carotene bleaching tests are expressed as percentage inhibition, FRAP test as μM Fe(II)/g. Differences within and between groups were evaluated by one-way ANOVA followed by a multicomparison Dunnett’s test α = 0.05): ****p < 0.0001, compared with the negative controls (control juice).

Figure 1 showed the results of the antioxidant activity of pasteurized, enriched juices. After pasteurization, a decrease in antioxidant activity of about 20% in the DPPH and ABTS test, about 15% in the FRAP test and 11.5% in the β-carotene bleaching test was observed. 

Generally, the increase in antioxidant activity was significantly higher in juices enriched with leaf extracts except for the ABTS test in which JPFI showed the highest activity.

In the DPPH assay, samples enriched with leaf extract showed a percentage of inhibition of 92.47% with an increase of 20% in antioxidant activity as compared to the control. An increase in DPPH radical scavenging activity of about 18% was observed with JPFI as compared with JPF. 

The ABTS antioxidant activity followed the trend JPFI > JPFE > JPFA, with inhibition values of 94.88, 93.31, and 91.14, respectively. Furthermore, results showed that JPFI showed an increase of 13% in antioxidant activity as compared to JPF. A similar value was found for JPFE with an increase of 12%. The potential of *C. × clementina* juice to inhibit lipid peroxidation was assessed using the β-carotene bleaching test. Generally, all enriched juice increased the antioxidant activity in comparison to JPF. The greatest activity was observed for JPFA, which increased its activity by 27 and 26% after 30 and 60 min of incubation, respectively. Interesting results were observed for JPFI, with increased activity about 25 and 23% after 30 and 60 min of incubation, respectively. Similar results were found for JPFE, which showed an increase of 22 and 21% after 30 and 60 min of incubation, respectively. 

FRAP values of 86.69, 85.01, and 72.87 μM Fe(II)/g for JPFA, JPFI, and JPFE, respectively, were found. In this assay, JPFA showed an increase of 17% FRAP compared to JF. A similar ability was observed for JPFI. The enrichment with 20% (mg/100 mL) peel extract did not cause a significant increase in FRAP.

*Pearson’s* correlation coefficient showed a positive correlation between TPC and DPPH and the β-carotene bleaching test after 30 and 60 min of incubation, with *r* values of 0.97, 0.99, and 0.98, respectively. Similar positive correlations were found between TPC, TFC, and the FRAP test, with *r* values of 0.87 and 0.88, respectively. TCC positively correlated with ABTS and the β-carotene bleaching test at 30 and 60 min of incubation, with r values of 0.95, 0.84 and 0.86, respectively. 

#### 3.10.3. Enzyme Inhibitory Activities 

**Figure 2:**
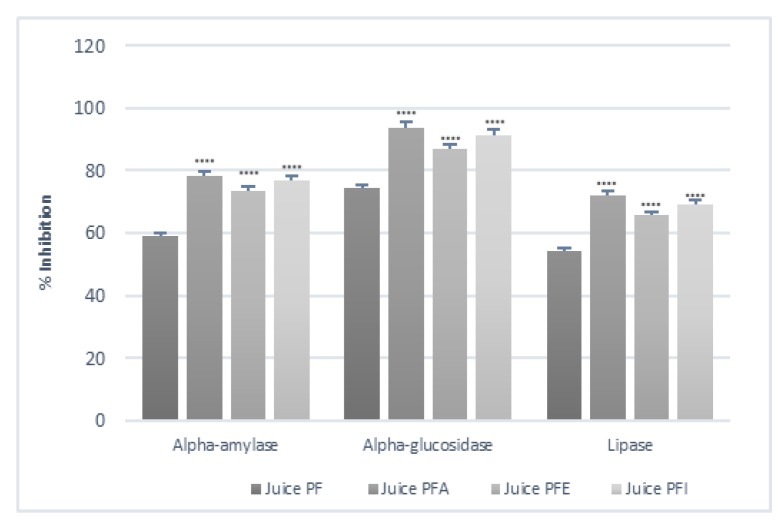
α-Amylase, α-glucosidase, and lipase inhibitory activity of *C. × clementina* pasteurized, enriched juice. JPF: pasteurized juice JF; JPFA: pasteurized, enriched juice JPF with 20% leaf extract; JPFE: pasteurized, enriched juice JF with 20% peel extract; JPFI: pasteurized, enriched juice JF with 20% peel + leaf extracts 1:1. Data are expressed as means ± S.D. (*n* = 3). Differences within and between groups were evaluated by one-way ANOVA followed by a multicomparison Dunnett’s test α = 0.05): ****p < 0.0001, compared with the negative controls (control juice).

The study of enzyme inhibitory activity of pasteurized juices was performed (Figure 2). The pasteurization process caused a decrease in enzyme inhibitory potential of about 24 and 20%, respectively, for α-amylase and α-glucosidase assays, and about 17% for lipase enzyme. No significant differences were observed for JPFA and JPFI in enzyme inhibition. In the α-glucosidase assay, JPFA induced an increase of 20% in hypoglycaemic activity followed by JPFI (13%) as compared to the control juice. Moreover, all pasteurized samples were able to inhibit the α-amylase enzyme in a concentration-dependent manner. JPFA induced an increase of 19% in hypoglycaemic activity followed by JPFI (17%) as compared to the control juice. 

A promising anti-obesity potential was found for JPFA, with inhibition of 72.25% followed by JFI with inhibition of 69.19%. A similar value was observed for JPFE. The pasteurized juice enriched with leaf extract showed an increase of 18% of the enzyme inhibitory activity as compared to the control juice. A positive *Pearson’s* correlation coefficient was found between TPC and all conducted tests (*r* = 1.00).

#### 3.10.4. Sensory Analysis

The sensory analysis was also carried out for enriched juice samples after pasteurization. The evaluation was done using a 9-point structured scale, with 9 being the best and 1 the worst product quality. Results of sensory analysis of clementine juice are reported in Table S7.

Based on the average ratings given by testers, the untreated enriched juices were more accepted compared to pasteurized juice. The pasteurized, peel-enriched juice again obtained the best scores for appearance, colour, and odour, while for other parameters, natural juice proved more attractive than enriched juice. Overall, the *C. × clementina* pasteurized, enriched juice was well accepted by the testers, with average scores above 7–8 for all concentrations. 

#### 3.10.5. PCA 

The projections of the observations on the first two principal component axes are shown in Figure S4. The accessions are distributed on the factor plane. These two coordinates represent 98% of the total variance (PC1 explained 58% of total variation, while PC2 explained 36% of total variation). The first component (PC1) was positively and strongly correlated with TFC, TPC, TCC, DPPH, ABTS, FRAP, α-amylase, and α-glucosidase.

The second component (PC2) was positively correlated with FRAP, β-carotene bleaching after 30 and 60 min incubation, and lipase. The scores plot was used to gain an overview of the similarities or differences among the juices (fresh juice JF, enriched fresh juices JFE, JFI, JFA, pasteurized juice JPF, and enriched pasteurized juices JPFE, JPFI, and JPFA). Figure S4 shows the spatial distribution of juice samples and the bioactive attributes associated with the juice samples, fresh and pasteurized. The spatial distributions of points relating to the juice samples formed separate clusters. Fresh juices and enriched fresh juices (JF, JFA, JFI, and JFE) were perceived as having better quality characteristics. The group of pasteurized juices (JPF, JPFA, JPFI, and JPFE) were characterized by a lower intensity of characteristics describing the first group. Antioxidant activity represented a common feature for JFE, JFI, and JFA. The PCA model showed that enriched fresh juices were characterized by higher TFC, TPC, and TCC contents as well as by higher DPPH, ABTS, FRAP, α-amylase, α-glucosidase, β-carotene bleaching 30 and 60 min incubation, and lipase activity in comparison to the pasteurized juices. Bearing in mind the above dependencies, the first principal component (PC1) may be interpreted as the measure of positive characteristics of fresh juices and enriched fresh juices.

## 4. Conclusions

The present work aimed to investigate a model for the reuse of *C. × clementina* by-products in order to propose their utilization for the development of functional beverages useful in the prevention of diseases associated with oxidative stress such as T2DM and obesity.

Juice enriched with leaf extract (JFA) was found to be richer in bioactive compounds, and this led to an improvement in antioxidant activity in the range from +20 to +30%. Moreover, JFA enhanced the inhibitory activity by up to +37 and +25% against α-glucosidase and α-amylase, respectively. The inhibitory activity of lipase increased by up to +17%. Despite that pasteurization reduces the content of bioactive compounds, this process did not significantly affect these positive results obtained with enriched juices.

Future studies will be conducted to evaluate the bioaccessibility and bioavailability of *C. × clementina* juice and derived products. The ability of *Citrus* flavanones to exert beneficial effects depends on their bioavailability, correlated with their chemical structure, food matrix, and host factors. A bioavailability study on hesperidin, the most abundant flavanone glycoside in *C. × clementina*, showed that, after intake, it was resistant to enzymatic breakdown in the stomach and small intestine and, therewith, mainly reached the colon intact [75]. Hesperidin, containing rutinose groups, is hydrolysed only in the distal part of the intestine and the colon by the gut microbiota bacteria, α-rhamnosidases, that remove rhamnose moiety, followed by the removal of glucose by β-glucosidases. This process contributes to the interindividual variability in the bioavailability of hesperidin.

In conclusion, based on the promising bioactivity of enriched juices and comforted by sensory analysis data, we propose *C. × clementina* by-products as a promising source of bioactive compounds useful for the formulation of functional drinks or foods for preventing diseases associated with oxidative stress, with particular reference to T2DM.

## Figures and Tables

**Table 1:** *C. × clementina* juice, peel, and leaf extracts.

Sample	Site of Collection	Abbreviation	Procedure
Juice			
	Cetraro	JD	Sqeezed
	Rosarno	JE	Squeezed
	Corigliano Calabro	JF	Squeezed
Peel extracts			
	Cetraro	BC1	Soxhlet extractor
		BC2	Ultrasound EtOH/H_2_O (80:20)
		BC3	Ultrasound EtOH
		BC4	Maceration EtOH/H_2_O (80:20)
		BC5	Maceration EtOH
		BC6	Hydrodistillation
	Rosarno	BR1	Soxhlet extractor
		BR2	Ultrasound EtOH/H_2_O (80:20)
		BR3	Ultrasound EtOH
		BR4	Maceration EtOH/H_2_O (80:20)
		BR5	Maceration EtOH
		BR6	Hydrodistillation
	Corigliano Calabro	BO1	Soxhlet extractor
		BO2	Ultrasound EtOH/H_2_O (80:20)
		BO3	Ultrasound EtOH
		BO4	Maceration EtOH/H_2_O (80:20)
		BO5	Maceration EtOH
		BO6	Hydrodistillation
Leaf extracts*			
	Corigliano Calabro	CO2	Ultrasound EtOH/H_2_O (80:20)

BC: Cetraro peel extracts; BR: Rosarno peel extracts; BO: Corigliano Calabro peel extracts; *Fresh leaf extract previously investigated [15].

**Table 2:** Enriched and pasteurized enriched juice.

Juice	Enrichment^
JFA	JF + 20% CO2
JFB	JF + 15% CO2
JFC	JF + 10% CO2
JFD	JF + 5% CO2
JFE	JF + 20% BC3
JFF	JF + 15% BC3
JFG	JF + 10% BC3
JFH	JF + 5% BC3
JFI	JF + 20% CO2 +BC3^^
JFL	JF + 15% CO2 +BC3^^
JFM	JF + 10% CO2 + BC3^^
JFN	JF + 5% CO2 + BC3^^
Pasteurized juices	
JPFA	JPF + 20% CO2
JPFE	JPF + 20% BC3
JPFI	JPF + 20% CO2 + BC3^^

^mg/100 mL; ^^extract added in a 1:1 ratio (mg/mg).

**Table 3:** *C. × clementina* juice content and nutritional constituents.

Parameters	JD	JE	JF	Sign.
Juice (pH)	3.54 ± 0.06^b^	3.47 ± 0.05^c^	3.72 ± 0.08^a^	**
Acidity (g/100 mL)	0.57 ± 0.03^c^	0.63 ± 0.05^b^	0.70 ± 0.03^a^	**
°Brix	10.10 ± 0.02^c^	10.41 ± 0.03^a^	10.24 ± 0.02^b^	**
Chroma value (C*)	30.48 ± 1.12^b^	28.22 ± 1.09^c^	34.04± 1.14^a^	**
Ascorbic acid (mg/100 mL)	65.12 ± 3.23^b^	64.48 ± 3.26^b^	66.34 ± 3.88^a^	**
Ash (g/100 g)	0.45 ± 0.04^b^	0.43 ± 0.03^b^	0.47 ± 0.05^a^	**
Fats (g/100 g)	0.17 ± 0.11^a^	0.16 ± 0.19^a^	0.15 ± 0.10^a^	ns
Protein (g/100 g)	0.80 ± 0.09^a^	0.83 ± 0.07^a^	0.82 ± 0.08^a^	ns
Fiber (g/100 g)	1.82 ± 1.12^a^	1.76 ± 1.04^b^	1.80 ± 1.14^a^	**
Carbohydrates (g/100 g)	12.31± 1.84^a^	12.10 ± 1.80^a^	12.01 ± 1.82^a^	ns
Energy kcal/100 g	54 ± 2.03^a^	53 ± 2.04^a^	52 ± 2.02^a^	ns
Phosphorus (mg/100 g)	20 ± 1.2^a^	21 ± 1.3^a^	22 ± 1.4^a^	ns
Potassium (mg/100 g)	181 ± 4.52^b^	178 ± 3.33^c^	183 ± 3.56^a^	**
Calcium (mg/100 g)	31 ± 1.24^a^	30 ± 1.27^a^	31 ± 1.25^a^	ns
Magnesium (mg/100 g)	12 ± 0.83^b^	12 ± 0.91^b^	13 ± 0.94^a^	**

Data are expressed as the mean ± standard deviation (SD) (*n* = 3). JD: juice from fruits collected in Cetraro; JE: juice from fruits collected in Rosarno; JF: juice from fruits collected in Corigliano Calabro. Differences were evaluated by one-way analysis of variance (ANOVA) completed with a multicomparison Tukey’s test. ** *p* < 0.05. Means in the same row with different small letters differ significantly (*p* < 0.05). Sign: significant. ns: not significant.

**Table 4:** Phytochemical contents of *C.× clementina* juice and peel extracts.

Sample	Yields	Total Phenol Content	Total Flavonoid Content	Total Carotenoid Content
Juice	(% L/Kg)	(mg CAE)/100 mL)	(mg QE)/100 mL)	(mg β-caroteneE)/100 mL)
JD	48.19 ± 6.07^b^	29.46 ± 1.11^b^	30.28 ± 1.17^b^	31.12 ± 1.19^b^
JE	47.13 ± 6.01^c^	17.58 ± 0.93^c^	18.16 ± 0.99^c^	18.23 ± 0.92^c^
JF	48.37 ± 6.42^b^	54.65 ± 2.92^a^	51.48 ± 2.84^a^	53.54 ± 2.89^a^
Sign.	**	**	**	**
Peels	(% g/g)	(mg CAE)/g FW)	(mg QE)/g FW)	(mg β-caroteneE)/g FW)
BC1	14.42 ± 1.41^a^	8.75 ± 0.83^a^	6.05 ± 0.67^a^	39.84 ± 3.47^a^
BC2	12.91 ± 1.24^c^	6.30 ± 0.64^c^	4.02 ± 0.42^d^	17.89 ± 1.76^c^
BC3	12.07 ± 1.23^e^	6.27 ± 0.68^c^	5.08 ± 0.58^b^	16.66 ± 1.63^d^
BC4	12.86 ± 1.27^c^	5.34 ± 0.56^f^	4.02 ± 0.44^d^	15.60 ± 1.57^g^
BC5	12.48 ± 1.20^d^	5.49 ± 0.51^e^	3.89 ± 0.46^e^	12.14 ± 1.25^i^
BR1	14.28 ± 1.42^b^	7.13 ± 0.78^b^	4.64 ± 0.47^c^	19.62 ± 1.98^b^
BR2	9.48 ± 0.97^n^	4.43 ± 0.45^h^	3.13 ± 0.33^l^	10.88 ± 1.11^m^
BR3	10.62 ± 1.01^i^	4.27 ± 0.43^l^	3.39 ± 0.31^h^	10.42 ± 1.05^o^
BR4	9.52 ± 0.94^n^	4.38 ± 0.44^i^	3.44 ± 0.36^h^	15.97 ± 1.69^f^
BR5	10.49 ± 1.00^l^	3.99 ± 0.45^m^	3.20 ± 0.34^i^	11.68 ± 1.11^l^
BO1	14.99 ± 1.47^a^	5.91 ± 0.56^d^	4.62 ± 0.49^c^	16.49 ± 1.68^e^
BO2	11.50 ± 1.11^e^	4.50 ± 0.41^g^	3.56 ± 0.34^g^	9.66 ± 0.92^q^
BO3	10.88 ± 1.04^g^	3.80 ± 0.38^o^	3.80 ± 0.36^f^	10.28 ± 1.05^p^
BO4	9.70 ± 0.93^m^	3.45 ± 0.36^p^	2.47 ± 0.22^n^	10.48 ± 1.02^n^
BO5	10.74 ± 1.07^h^	3.85 ± 0.37^n^	2.78 ± 0.28^m^	13.60 ± 1.37^h^
Sign.	**	**	**	**

Data represent means ± SD (standard deviation) (*n* = 3). Differences were evaluated by one-way analysis of variance (ANOVA) completed with a multicomparison Tukey’s test. ** *p* < 0.05. Means in the same column with different small letters differ significantly (*p* < 0.05). Sign: significant. ns: not significant.

**Table 5:** The main components of *C. × clementina* peel essential oils.

Compounds	RI^1^	Relative Amount (%)			I.M^2^	Sign.
		BC6	BR6	BO6		
α-Pinene	938	1.10 ± 0.12^c^	1.55 ± 0.21^b^	3.13 ± 0.33^a^	1,2,3	**
Sabinene	973	2.55 ± 0.70^a^	0.97 ± 0.12^c^	1.52 ± 0.21^b^	1,2,3	**
β-Pinene	980	tr	tr	tr	1,2,3	**
Myrcene	993	3.56 ± 0.31^c^	4.94 ± 0.46^b^	9.10 ± 0.91^a^	1,2,3	**
+/- Limonene	1030	75.11± 4.55^b^	83.09 ± 5.12^a^	61.31 ± 4.02^c^	1,2,3	**
(E)- β-Ocimene	1049	3.31 ± 0.37^a^	nd	nd	1,2	**
γ-Terpinene	1057	0.33 ± 0.03^a^	tr	0.32 ± 0.03^a^	1,2,3	**
Terpinolene	1086	tr	tr	0.30 ± 0.03^a^	1,2,3	**
Linalool	1098	5.30 ± 0.55^b^	3.29 ± 0.36^c^	6.64 ± 0.61^a^	1,2,3	**
Nonanal	1100	1.84 ± 0.23^b^	1.62 ± 0.24^c^	3.64 ± 0.34^a^	1,2	**
Citronellal	1148	0.40 ± 0.04^b^	tr	0.75 ± 0.07^a^	1,2	**
Terpinen-4-ol	1176	0.56 ± 0.05^b^	0.20 ± 0.02^c^	0.88 ± 0.08^a^	1,2	**
α-Terpineol	1189	0.47 ± 0.04^b^	0.49 ± 0.04^b^	1.55 ± 0.12^c^	1,2,3	**
Decanal	1205	1.05 ± 0.10^b^	1.04 ± 0.10^b^	2.20 ± 0.21^a^	1,2	**
trans-Caryophyllene	1415	tr	tr	tr	1,2,3	**
trans-β-Farnesene	1441	tr	tr	0.35 ± 0.03^a^	1,2	**
α-Humulene	1454	tr	tr	tr	1,2,3	**
δ-cadinene	1526	0.31 ± 0.02^a^	0.22 ± 0.02^c^	0.26 ± 0.02^b^	1,2	**
β-Sinensal	1697	0.29 ± 0.02^a^	tr	0.13 ± 0.02^b^	1,2	**
α-Sinensal	1750	0.70 ± 0.07^a^	0.37 ± 0.04^c^	0.45 ± 0.05^b^	1,2	**
Total identified		96.88	97.78	92.53		

BC6: Peel EO from Cetraro; BR6: Peel EO from Rosarno; BO6: Peel EO from Corigliano Calabro. Data are reported as the mean ± standard deviation (*n* = 3). ^1^RI: Retention indices on the HP 5MS column. ^2^IM, identification method: 1—comparison of retention times; 2—comparison of mass spectra with MS libraries, 3—comparison with authentic compounds; tr: trace (<0.1%). Differences were evaluated by one-way analysis of variance (ANOVA) completed with a multicomparison Tukey’s test. **p < 0.05. Means in the same row with different small letters differ significantly (*p* < 0.05). nd: not detected.

**Table 6:** HPLC analysis of selected markers of *C. × clementina* juice.

Selected Markers	JD	JE	JF	Sign.
Apigenin	0.06 ± 0.04^c^	0.05 ± 0.03^b^	0.09 ± 0.08^a^	**
Caffeic acid	7.48 ± 1.61^b^	3.65 ± 1.30^c^	8.52 ± 1.81^a^	**
Chlorogenic acid	2.59 ± 1.22^a^	2.06 ± 1.20^b^	2.56 ± 1.26^a^	**
Didymin	3.85 ± 1.26^c^	4.17 ± 1.33^b^	5.51 ± 0.04^a^	**
Eriocitrin	1.84 ± 0.19^c^	2.33 ± 1.34^a^	0.91 ± 0.11^b^	**
Gallic acid	1.02 ± 0.09^b^	0.62 ± 0.04^c^	1.67 ± 0.18^a^	**
Hesperidin	81.08 ± 4.94^a^	40.06 ± 3.04^c^	65.3 ± 3.54^b^	**
Naringin	1.73 ± 0.18^c^	2.12 ± 1.19^b^	3.14 ± 1.32^a^	**
Narirutin	8.50 ± 1.83^a^	6.25 ± 1.32^c^	7.88 ± 1.81^b^	**
Neoeriocitrin	2.69 ± 0.72^c^	3.14 ± 1.04^b^	3.41 ± 1.39^a^	**
Neohesperidin	110.63 ± 5.52^b^	80.26 ± 4.83^c^	112.32 ± 5.63^a^	**
Nobiletin	0.06 ± 0.01^c^	0.12 ± 0.01^b^	0.14 ± 0.01^a^	**
*p*-Coumaric acid	7.67 ± 1.69^b^	6.47 ± 1.58^c^	8.29 ± 1.72^a^	**
Poncirin	1.52 ± 0.19^c^	1.74 ± 0.21^b^	2.63 ± 0.51^a^	**
Protocatechuic acid	1.02 ± 0.74^b^	0.62 ± 0.07^c^	1.67 ± 0.96^a^	**
Quercetin	0.42 ± 0.03^b^	0.56 ± 0.05^c^	1.32 ± 0.13^a^	**
Sinensetin	0.005 ± 0.01^b^	0.006 ± 0.01^b^	0.01 ± 0.03^a^	**
Tangeretin	0.06 ± 0.07^a^	0.05 ± 0.06^b^	0.07 ± 0.08^a^	**
Vanillic acid	0.69 ± 0.07^b^	2.16 ± 1.30^a^	0.43 ± 0.03^c^	**
∑ Indentified phenols	232.92	156.44	226.01	

JD: juice from fruits collected in Cetraro; JE: juice from fruits collected in Rosarno; JF: juice from fruits collected in Corigliano Calabro. Data are expressed as the mean ± standard deviation (SD) (*n* = 3). Differences were evaluated by one-way analysis of variance (ANOVA) completed with a multicomparison Tukey’s test. ** *p* < 0.05. Means in the same row with different small letters differ significantly (*p* < 0.05). Results are expressed as mg/100 mL juice. Sign: significant.

**Table 7:** HPLC–DAD of *C.× clementina* peel extracts (mg/100g FW).

Sample	Tangeretin	Sinensetin	Luteolin	Quercetin	Quercetin-3-*O*-glucoside	Poncirin	Hesperidin	Neoeriocitrin	Eriocitrin	Caffeic Acid
BC1	7.47 ± 0.78^cd^	32.86 ± 3.23^c^	6.17 ± 0.68^d^	8.98 ± 0.93^a^	11.26 ± 1.27^a^	1.98 ± 0.12^b^	778.70 ± 15.91^d^	4.89 ± 0.57^a^	3.75 ± 0.33^a^	0.22± 0.02^h^
BC2	7.92 ± 0.80^bc^	29.36 ± 2.94^f^	5.16 ± 0.50^h^	7.98 ± 0.72^b^	8.49 ± 0.96^d^	0.57 ± 0.05^g^	1007.86 ± 8.12^b^	3.29 ± 0.32^b^	2.12 ± 0.21^b^	6.98 ± 0.64^d^
BC3	6.57 ± 0.77^fg^	27.07 ± 2.76^g^	4.05 ± 0.47^m^	6.22 ± 0.61^g^	6.61 ± 0.68^h^	2.29 ± 0.34^a^	1093.36 ± 8.13^a^	2.43 ± 0.26^d^	1.56 ± 0.14^c^	0.15 ± 0.01^h^
BC4	5.43 ± 0.53^l^	26.22 ± 2.62^h^	5.02 ± 0.54^i^	7.72 ± 0.77^c^	8.20 ± 0.83^e^	2.53 ± 0.28^a^	228.63 ± 8.13^l^	2.99 ± 0.37^c^	2.02 ± 0.26^b^	6.40 ± 0.61^e^
BC5	7.19 ± 0.79^def^	32.56 ± 3.21^c^	5.39 ± 0.05^g^	7.95 ± 0.08^b^	8.45 ± 0.08^d^	2.65 ± 0.02^a^	238.53 ± 8.22^i^	0.68 ± 0.077^e^	3.62 ± 0.36^a^	0.20 ± 0.02^h^
BR1	8.31 ± 0.85^b^	37.99 ± 3.74^a^	5.53 ± 0.53^f^	6.86 ± 0.64^e^	7.29 ± 0.71^g^	1.33 ± 0.17^de^	977.23 ± 18.11^c^	nd	nd	nd
BR2	6.47 ± 0.66^gh^	25.59 ± 2.11^i^	3.77 ± 0.48^n^	8.02 ± 0.84^b^	7.27 ± 0.73^g^	1.35 ± 0.18^de^	558.14 ± 15.24^f^	nd	nd	5.32 ± 0.51^f^
BR3	7.0± 0.77^ef^	26.39± 2.63^h^	3.53 ± 0.39^o^	5.78 ± 0.52^h^	nd	1.39 ± 0.17^cd^	667.18 ± 15.54^e^	nd	nd	nd
BR4	9.6 ± 0.97^a^	37.2 ± 3.40^b^	8.2 ± 0.87^c^	nd	nd	nd	100.26 ± 8.15^o^	nd	nd	8.99 ± 0.94^b^
BR5	7.37 ± 0.76^de^	31.41± 3.12^d^	5.74 ± 0.57^e^	nd	nd	n.d	173.52 ± 8.13^m^	nd	nd	10.87 ± 1.22^a^
BO1	6.75 ± 0.62^gf^	31.51± 3.14^d^	6.11 ± 0.63^d^	7.13 ± 0.77^d^	7.58 ± 0.71^f^	1.51 ± 0.19^cd^	243.98 ± 10.21^hi^	nd	nd	2.68 ± 0.31^g^
BO2	6.06 ± 0.61^hi^	30.74 ± 3.07^e^	4.72 ± 0.43^l^	7.76 ± 0.88^c^	6.37± 0.66^i^	1.21 ± 0.17^ef^	364.52 ± 11.33^g^	nd	0.73 ± 0.07^e^	8.0 ± 0.82^c^
BO3	6.2 ± 0.63^hi^	19.56 ± 2.19^m^	3.02 ± 0.32^p^	7.7 ± 0.87^c^	6.66 ± 0.63^h^	1.61 ± 0.17^c^	253.8 ± 10.15^h^	nd	0.88 ± 0.08^d^	nd
BO4	5.8 ± 0.68^li^	25.7 ± 2.56^i^	8.58 ± 0.94^a^	7.07 ± 0.72^d^	10.54 ± 1.26^b^	1.08 ± 0.17^f^	155.28 ± 8.12^n^	nd	nd	nd
BO5	5.55 ± 5.01^l^	21.2 ± 2.13^l^	8.36 ± 0.87^b^	6.51 ± 0.65^f^	9.65 ± 0.92^c^	1.05 ± 0.18^f^	179.42 ± 8.16^m^	nd	0.78 ± 0.08^d^	nd
Sign.	**	**	**	**	**	**	**	**	**	**

Data represent means ± SD (standard deviation) (*n* = 3). Differences were evaluated by one-way analysis of variance (ANOVA) test completed with a multicomparison Tukey’s test. ** *p* < 0.05. Means in the same column with different small letters differ significantly (*p* < 0.05). Sign: significant; nd: not detected.

**Table 8:** Antioxidant activity of *C. × clementina* juice.

Sample	DPPH Test IC_50_ (μg/mL)	ABTS Test IC_50_ (μg/mL)	β-Carotene Bleaching test IC_50_ (μg/mL)		FRAP Test μM Fe (II)/g	RACI Values
			t 30 min	t 60 min		
JD	82.43 ± 2.96^****^	33.63 ± 2.01^****^	25.90%	27.50%	2.8 ± 0.96^****^	−0.13
JE	84.02 ± 2.92^****^	40.32 ± 2.54^****^	27.50%	28.47%	3.01 ± 0.98^****^	0.72
JF	81.13 ± 2.73^****^	24.82 ± 1.96^****^	31.33%	34.20%	5.70 ± 1.00^****^	−0.59
*Positive control*						
Ascorbic acid	5.01 ± 0.80	1.72 ± 0.06				
Propyl gallate			0.09 ± 0.004	0.09 ± 0.004		
BHT					63.23 ± 4.31	

JD: juice from fruits collected in Cetraro; JE: juice from fruits collected in Rosarno; JF: juice from fruits collected in Corigliano Calabro. Data are expressed as means ± S.D. (*n* = 3). Differences within and between groups were evaluated by one-way ANOVA followed by a multicomparison Dunnett’s test (α = 0.05): ****p < 0.0001, compared with the positive controls.

**Table 9:** Antioxidant activity of *C. × clementina* peels.

Sample	DPPH Test IC_50_ (µg/mL)	ABTS Test IC_50_ µg/mL)	β-Carotene Bleaching Test IC_50_ (µg/mL)		FRAP μM Fe (II)/g	RACI
			t = 30 min	t = 60 min		
BC1	105.66 ± 4.01^****^	14.82 ± 1.22^****^	32.52 ± 1.93^****^	11.22 ± 1.08^****^	23.51 ± 1.77^****^	−0.55
BC2	52.58 ± 2.32^****^	8.22 ± 0.84^****^	75.03 ± 2.77^****^	22.86 ± 1.93^****^	28.48 ± 1.83^****^	−0.37
BC3	45.79 ± 2.14^****^	15.06 ± 1.62	16.47 ± 1.85^****^	12.53 ± 1.17^****^	34.28 ± 1.95^****^	−0.59
BC4	117.86 ± 4.07^****^	17.54 ± 1.91^****^	90.99 ± 3.93^****^	12.53 ± 1.26^****^	26.26 ± 1.77^****^	−0.43
BC5	140.39 ± 4.35^****^	21.03 ± 1.79^****^	18.26 ± 1.81^****^	10.47 ± 1.08^****^	23.19 ± 1.63^****^	0.02
BC6	308.55 ± 6.12^****^	24.13 ± 2.03^****^	91.92 ± 3.98	47.22 ± 2.21^****^	6.13 ± 0.61^****^	0.51
BR1	68.13 ± 2.43^****^	15.21 ± 1.23^****^	36.84 ± 1.94^****^	11.45 ± 1.16^****^	30.97 ± 1.97^****^	−0.46
BR2	81.26 ± 3.41^****^	10.97 ± 1.05^****^	68.75 ± 2.96^****^	58.52 ± 2.57^****^	25.78 ± 1.73^****^	−0.05
BR3	113.17 ± 4.03	9.47 ± 0.97^****^	8.78 ± 0.83^****^	19.38 ± 1.83^****^	54.95 ± 2.14	−0.51
BR4	83.14 ± 3.61^****^	20.25± 1.94^****^	96.8 ± 3.18^****^	89.39 ± 3.82^****^	40.46 ± 2.47^****^	−0.10
BR5	125.37 ± 4.11^****^	11.76 ± 1.13^****^	43.22 ± 2.11^****^	72.4 ± 2.71^****^	21.13 ± 1.03^****^	1.20
BR6	370.3 ± 6.74^****^	26.30 ± 2.07^****^	55.7 ± 2.53^****^	10.37 ± 0.93^****^	23.91 ± 1.74^****^	0.48
BO1	259.57 ± 5.31^****^	31.50 ± 2.11^****^	71.00 ± 2.64^****^	11.41 ± 0.94^****^	6.48 ± 0.62^****^	0.20
BO2	174.17 ± 4.43^****^	28.37 ± 2.07^****^	72.78 ± 2.75^****^	64.15 ± 2.76^****^	21.26 ± 1.71^****^	0.63
BO3	212.65 ± 3.83^****^	15.21 ± 1.13^****^	87.98 ± 2.83	39.09 ± 2.17^****^	27.39 ± 1.37^****^	−0.18
BO4	169.48 ± 4.87^****^	14.05 ± 1.25^****^	87.98 ± 3.61^****^	39.09 ± 2.16^****^	26.1 ± 1.83^****^	0.15
BO5	212.65 ± 4.52^****^	8.22 ± 0.89^****^	58.33 ± 2.59^****^	12.90 ± 0.95^****^	27.39 ± 1.85^****^	−0.21
BO6	333.7 ± 6.01^****^	18.31 ± 1.91^****^	61.8 ± 2.62^****^	11.78 ± 1.02^****^	25.38 ± 1.71^****^	0.25
*Positive* *control*						
Ascorbic acid 5.0 ± 0.8		1.7 ± 0.06				
Propyl gallate			0.0 9 ± 0.004	0.09 ± 0.004		
BHT					82.43± 1.52	

Data are expressed as means ± S.D. (*n* = 3). Differences within and between groups were evaluated by one-way ANOVA followed by a multicomparison Dunnett’s test α = 0.05): ****p < 0.0001, compared with the positive controls.

**Table 10:** Hypoglycaemic and hypolipidemic effects of *C. × clementina* juice, peel polar extracts, and essential oils.

Sample	α-Amylase IC_50_ (μg/mL)	α-Glucosidase IC_50_ (μg/mL)	Lipase IC_50_ (μg/mL)
JD	189.81 ± 2.09****	89.37 ± 2.07****	192.14 ± 2.47****
BC1	210.68 ± 4.95****	141.32 ± 4.38****	186.14 ± 4.24****
BC2	132.00 ± 4.22****	152.15 ± 4.47****	145.59 ± 3.71****
BC3	79.73 ± 3.64****	71.97 ± 2.61****	112.06 ± 3.64****
BC4	146.89 ± 4.39****	101.91 ± 3.92****	186.54 ± 4.2****
BC5	258.13 ± 5.18****	126.75 ± 4.14****	189.37 ± 4.31****
BC6	228.35 ± 4.94****	225.35 ± 4.90****	181.48 ± 4.04****
JE	194.33 ± 2.15****	103.43 ± 2.43****	197.69 ± 2.68****
BR1	154.77 ± 4.42****	152.15 ± 4.42****	171.12 ± 3.82****
BR2	146.02 ± 4.34****	202.07 ± 4.80***	181.37 ± 4.05****
BR3	237.98 ± 4.97****	239.73 ± 5.16****	191.91 ± 4.80****
BR4	128.50 ± 4.12****	256.07 ± 5.27****	174.15 ± 3.81****
BR5	181.93 ± 4.36****	197.69 ± 4.85****	179.63 ± 3.92****
BR6	185.43 ± 4.11****	287.91 ± 5.24****	182.74± 4.13****
JF	139.89 ± 1.81****	67.19 ± 1.31****	179.32 ± 2.19****
BO1	207.33 ± 4.83****	130.25 ± 4.25****	173.42 ± 4.02****
BO2	167.91 ± 4.55****	129.37 ± 4.27****	142.06 ± 3.85****
BO3	160.91 ± 4.52****	143.39 ± 4.45****	132.37 ± 3.56****
BO4	280.03 ± 5.23****	138.13 ± 4.35****	165.18 ± 3.95****
BO5	186.13 ± 4.60****	224.84 ± 2.97****	179.83 ± 4.01****
BO6	252.00 ± 5.12****	260.76 ± 5.13****	198.36 ± 4.83****
Positive control			
Acarbose	50.01 ± 0.92	35.52 ± 1.23	
Orlistat			37.63 ± 1.01

Data are expressed as means ± S.D. (*n* = 3). Acarbose was used as a positive control in α -amylase and α-glucosidase tests. Orlistat was use as a positive control in lipase test. Differences within and between groups were evaluated by one-way ANOVA followed by a multicomparison Dunnett’s test (α = 0.05): ****p < 0.0001, compared with the positive control.

**Table 11:** Antioxidant activity of *C. × clementina-*enriched juice.

Sample	DPPH Test % Inhibition	ABTS Test % Inhibition	β-carotene Bleaching Test % Inhibition		FRAP μM Fe (II)/g
			t = 30 min	t = 60 min	
JFA	122.71 ± 2.74****	115.46 ± 2.21****	52.47 ± 1.26****	64.56 ± 1.38****	100.43 ± 2.47****
JFB	118.23 ± 2.61****	111.78 ± 2.19****	50.32 ± 1.20****	60.29 ± 1.33****	98.36 ± 1.73****
JFC	105.21 ± 2.55****	110.77± 2.17****	49.83 ± 1.22****	53.24 ± 1.27****	97.95 ± 1.65****
JFD	97.78 ± 2.51*	107.64 ± 1.79****	43.89 ± 1.14****	49.73 ± 1.26****	91.93 ± 1.67****
JFE	108.21 ± 2.83****	112.32 ± 1.93****	49.52 ± 1.21****	59.37 ± 1.28****	89.58 ± 1.63****
JFF	101.77 ± 2.34****	110.84 ± 1.95****	48.43 ± 1.20****	57.22 ± 1.21****	88.56 ± 1.61***
JFG	98.40 ± 2.26**	109.77± 1.97****	46.14 ± 1.18****	45.22 ± 1.16****	86.33 ± 1.60*
JFH	95.15 ± 2.21^ns^	107.43 ± 1.97****	41.59 ± 1.14****	39.47 ± 1.17***	83.37 ± 1.57^ns^
JFI	106.23 ± 2.55****	118.87± 1.94****	49.73 ± 1.33****	62.63 ± 1.33****	99.28 ± 1.75****
JFL	101.78 ± 2.37****	113.92 ± 1.93****	49.02 ± 1.28****	60.14 ± 1.32****	96.72 ± 1.67****
JFM	98.57 ± 2.34**	109..57 ± 1.83****	48.61 ± 1.11****	53.62 ± 1.31****	87.04 ± 1.53**
JFN	96.57 ± 2.20^ns^	97.21 ± 1.65****	42.16 ± 1.13****	48.23 ± 1.26****	84.21 ± 1.54^ns^
*Negative control*					
JF	94.68 ± 2.2	96.87 ± 0.06	34.20 ± 1.06	34.25 ± 1.08	82.43± 1.52

Data are expressed as means ± S.D. (*n* = 3). Differences within and between groups were evaluated by one-way ANOVA followed by a multicomparison Dunnett’s test α = 0.05): ****p < 0.0001, ***p < 0.001, **p < 0.01, *p < 0.1 compared with the positive controls. ns: not significant.

**Table 12:** α-Amylase, α-glucosidase and lipase inhibitory activity (%) of *C.× clementina-*enriched juice.

Sample	α-Amylase	α-Glucosidase	Lipase
JFA	120.53 ± 2.91****	134.36 ± 3.01****	89.36 ± 2.22****
JFB	117.83 ± 2.82****	122.12 ± 2.95****	81.41 ± 2.12****
JFC	111.01 ± 2.69****	118.73 ± 2.90****	77.71 ± 2.03***
JFD	98.46 ± 2.63^ns^	112.52 ± 2.60****	75.48 ± 2.02****
JFE	112.64 ± 2.61****	110.68 ± 2.59****	83.11 ± 2.33****
JFF	102.34 ± 2.53***	106.76 ± 2.52****	78.93 ± 2.06****
JFG	99.83 ± 2.68^ns^	102.41 ± 2.54****	76.24 ± 2.08***
JFH	98.12 ± 2.64^ns^	99.34 ± 2.58***	73.56 ± 2.02***
JFI	118.67 ± 2.80****	118.12 ± 2.90****	86.42 ± 2.28****
JFL	116.43 ± 2.71****	115.43 ± 2.60****	81.02 ± 2.18****
JFM	110.91 ± 2.62****	106.24 ± 2.53****	78.93 ± 1.98****
JFN	98.03 ± 2.61^ns^	100.01 ± 2.50****	74.57 ± 1.91***
Negative control			
JF	96.08 ± 2.42	97.05 ± 1.23	72.36 ± 1.01

Data are expressed as means ± S.D. (*n* = 3). JF was used as a negative control. Differences within and between groups were evaluated by one-way ANOVA followed by a multicomparison Dunnett’s test (α = 0.05): ****p < 0.0001, ***p < 0.001, compared with the positive control. ns: not significant.

**Table 13:** Results of sensory analysis of enriched juice.

Sample	Appearance	Colour	Odour	Aroma	Sweetness	Aidity	Astringency	Mouthfeel
JF	8.12^ed^	8.52^ab^	8.01^c^	8.14^ab^	8.23^a^	7.98^a^	8.03^a^	8.26^a^
JFA	8.01^f^	8.23^f^	7.87^e^	7.96^e^	8.01^c^	7.93^a^	7.86^de^	7.98^i^
JFB	8.04^f^	8.28^ef^	7.93^d^	7.98^de^	8.12^abc^	7.95^a^	7.89^cd^	8.01^h^
JFC	8.08^e^	8.33^def^	7.98^d^	8.02^dc^	8.14^abc^	7.96^a^	7.91^cb^	8.15^cb^
JFD	8.11^e^	8.49^ab^	8.00^c^	8.09^b^	8.18^ab^	7.98^a^	7.96^ab^	8.17^b^
JFE	8.32^a^	8.55^a^	8.11^a^	8.03^c^	8.03^bc^	7.95^a^	7.87^de^	8.06^fg^
JFF	8.30^a^	8.54^a^	8.07^b^	8.09^b^	8.14^abc^	7.95^a^	7.92^cb^	8.10^cde^
JFG	8.25^b^	8.53^b^	8.06^b^	8.12^ab^	8.17^ab^	7.96^a^	7.98^b^	8.12^cd^
JFH	8.22^b^	8.52^ab^	8.02^c^	8.16^ab^	8.21^a^	7.97^a^	8.02^a^	8.16^cb^
JFI	8.11^ed^	8.38^cde^	8.07^b^	8.10^b^	8.05^abc^	7.94^a^	7.85^e^	8.04^gh^
JFL	8.13^cd^	8.42^bcd^	8.04^b^	8.12^ab^	8.13^abc^	7.96^a^	7.89^cd^	8.08^ef^
JFM	8.14^c^	8.46^abcd^	8.05^b^	8.13^ab^	8.16^abc^	7.98^a^	7.92^cb^	8.12^de^
JFN	8.12^ed^	8.52^ab^	8.00^c^	8.15^a^	8.20^a^	7.98^a^	7.98^b^	8.13^bcd^
Sign.	**	**	**	**	**	**	**	**

Data represent means ± SD (standard deviation) (*n* = 3). Differences were evaluated by one-way analysis of variance (ANOVA) completed with a multicomparison Tukey’s test. **p < 0.05. Means in the same column with different small letters differ significantly (*p* < 0.05). Sign: significant

## Supplementary Materials

The following are available online at https://www.mdpi.com/2076-3921/9/4/298/s1, Figure S1: PCA of *C. × clementina* juice, Figure S2: PCA of *C. × clementina* peel extracts, Figure S3: Phytochemical content in pasteurized juices, Figure S4: PCA enriched juice samples, Table S1: Calibration curves, detection limits (LOD), quantification limits (LOQ) of analytical method for determination of phenolic compound in *Citrus × clementina* samples, Table S2: Calibration curves, detection limits (LOD), quantification limits (LOQ) of analytical method for determination of coumarins in *Citrus × clementina* samples, Table S3: Fruit quality characteristics, Table S4: Positive *Pearson’s* correlation between phenolic compound contents in investigated juice and activity, Table S5: Enriched juice quality parameters, Table S6: Pasteurized enriched juice quality parameters, Table S7: Results of sensory analysis of pasteurized, enriched clementine juice.
