# Supplementary material for: Citrus × Clementina Hort. Juice Enriched with Its By-Products (Peels and Leaves): Chemical Composition, In Vitro Bioactivity, and Impact of Processing
Source: Antioxidants (Basel). 2020 Apr 3;9(4):298. doi: 10.3390/antiox9040298 (PMC7222210; doi:10.3390/antiox9040298)
Supplement: Supplementary file 1 [file antioxidants-09-00298-s001.pdf]

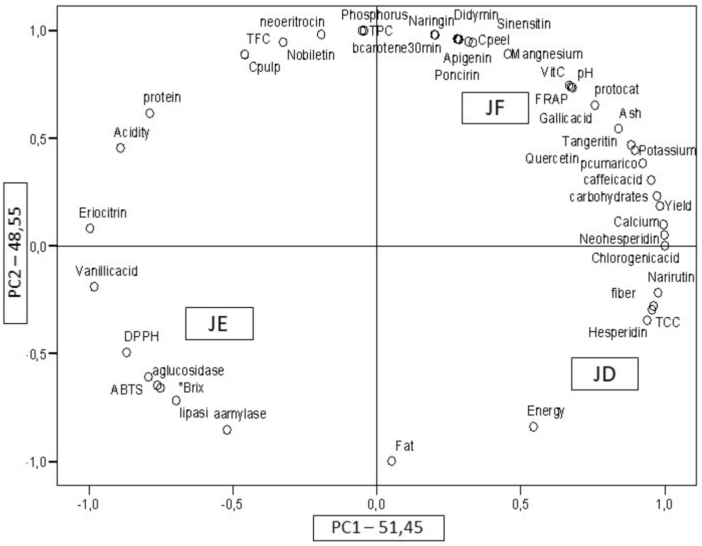

Figure S1. PCA analysis of *C.* × *clementina* juice

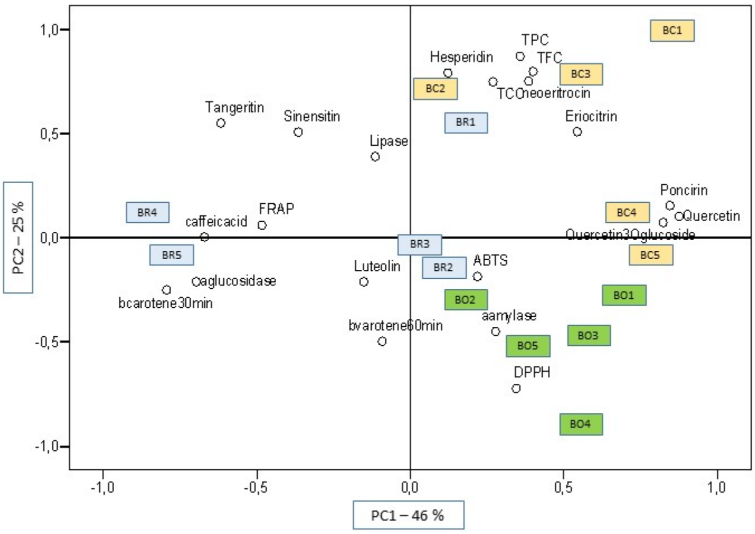

Figure S2. PCA analysis of *C.* × *clementina* peels extracts

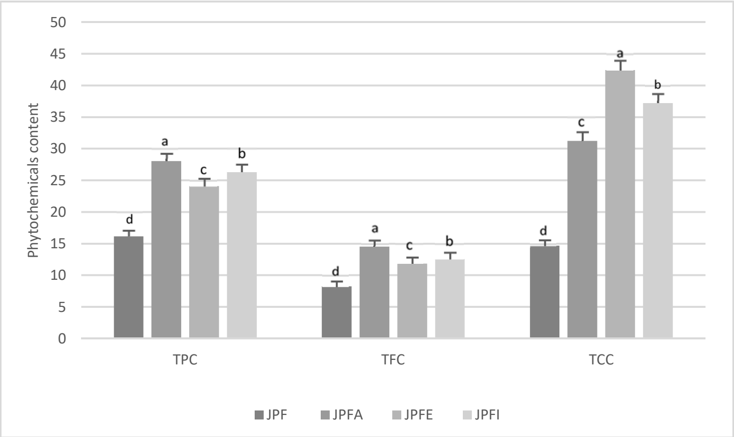

Data represent means ± SD (standard deviation) (n = 3). Differences were evaluated by one-way analysis of variance (ANOVA) test completed with a multicomparison Tukey's test. **p< 0.05. Means in the same column with different small letters differ significantly (p< 0.05).

Figure S3. Phytochemicals content in pasteurized juices

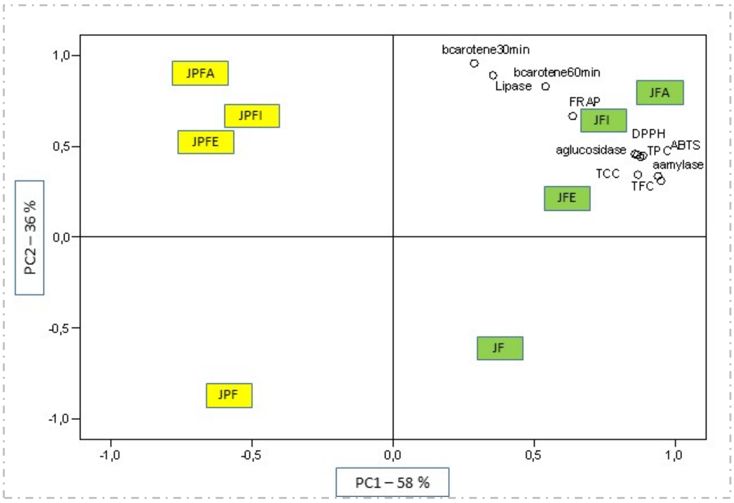

Figure S4. PCA enriched juice samples

Table S1. Calibration curves, detection limits (LOD), quantification limits (LOQ) of analytical method for determination of phenolic compounds in *Citrus × clementina* samples

STANDARD COMPOUNDS	Specificity		LOD[a]	LOQ[b]	Precision (RDS %)[d]
	Equation	r^2	mg/L	mg/L	Repeatability
Apigenin	y = 13.331x - 54.145	0.9982	0.001	0.012	0.03
Caffeic acid	y = 235.28x + 9.0586	0.9994	0.003	0.010	0.06
Chlorogenic acid	y = 125.72x - 4.4446	0.9994	0.004	0.025	0.04
Didymin	y = 83.401x + 2.1416	0.9999	0.003	0.018	0.02
Eriocitrin	y = 71.885x -0.2384	0.9999	0.001	0.024	0.08
Gallic acid	y = 39.423x -14.999	0.9994	0.001	0.014	0.02
Hesperidin	y = 15.013x + 1.1586	0.9998	0.001	0.012	0.02
Luteolin	y = 154.38x + 9.7542	0.9997	0.001	0.012	0.04
Naringin	y = 76.64x + 12.726	0.9993	0.002	0.027	0.05
Narirutin	y = 81.047x + 3.762	0.9993	0.003	0.014	0.05
Neoeriocitrin	y = 79.482x + 5.9704	0.9992	0.002	0.015	0.04
Neohesperidin	y = 143.95x + 0.5859	0.9999	0.003	0.018	0.02
Nobiletin	y = 13.275x + 6.2888	0.9999	0.004	0.017	0.05
p-Coumaric acid	y = 173.56x + 7.4967	0.9994	0.001	0.014	0.02
Poncirin	y = 190.94x - 1.3754	0.9999	0.002	0.022	0.01
Protocatechuic acid	y = 144.54x +4.3734	0.9994	0.004	0.018	0.03
Quercetin	y = 138.33x - 342.07	0.9991	0.002	0.027	0.03
Quercetin-3-O glucoside	y = 134.99x - 354.9	0.9989	0.002	0.018	0.04
Sinensetin	y = 107.07x + 16.575	0.9997	0.001	0.010	0.04
Tangeritin	y = 168x + 3.2604	0.9992	0.002	0.032	0.06
Vanillic acid	y = 134.01x + 43.634	0.9996	0.001	0.014	0.02

[a] Defined as the signal height at a signal/noise ratio S/N = 3.
[b] Defined as the signal height at a signal/noise ratio S/N = 10.
[c] Precision was expressed as the rsd % of N=3 determination

Table S2. Calibration curves, detection limits (LOD), quantification limits (LOQ) of analytical method for determination of coumarins in *Citrus × clementina* samples

STANDARD COMPOUNDS	Specificity		LOD[a]	LOQ[b]	Precision (RDS %)[d]
	Equation	r^2	mg/L	mg/L	Repeatability
Umbelliferone	y = 1204.2x + 90.928	0.9946	0.001	0.003	0.02
Isopimpinellin	y = 2657.5x + 987.46	0.9930	0.06	0.21	0.04
Bergapten	y = 1270.1x + 99.83	0.9905	0.02	0.06	0.03

[a] Defined as the signal height at a signal/noise ratio S/N = 3.
[b] Defined as the signal height at a signal/noise ratio S/N = 10.
[c] Precision was expressed as the rsd % of N=3 determination

Table S3. Fruit quality characteristics

Parameters	Cetraro (D)	Rosarno (E)	Corigliano Calabro (F)
Fruit weigh (g)	87.19 ± 3.21^{d}	109.67 ± 5.91^{c}	116.61 ± 9.13^{b}
Equatorial diameter (cm)	4.67 ± 0.76^{c}	4.83 ± 0.79^{b}	4.55 ± 0.74^{d}
Longitudinal diameter(cm)	4.54 ± 0.81^{c}	6.23 ± 0.85^{b}	6.72 ± 0.98^{a}
Fruit firmness (g/0.5 cm^2)	410.19 ± 12.99^{d}	412.28 ± 14.56^{c}	420.92 ± 14.21^{b}
Peels thickness(mm)	11.12 ± 0.28^{d}	11.22 ± 0.27^{c}	12.31 ± 0.40^{a}
Total seeds per fruit	4.25 ± 0.24^{c}	5.54 ± 0.32^{b}	4.14 ± 0.24^{c}

Data are expressed as mean ± standard deviation (SD) (n= 3). Differences were evaluated by one-way analysis of variance (ANOVA) test completed with a multicomparison Tukey's test. ** $p< 0.05$. Means in the same column with different small letters differ significantly ($p< 0.05$).

Table S4. Positive *Pearson*'s correlation between phenolic compounds content in investigated juice and activity

	**DPPH test**	**ABTS test**	**β-carotene bleaching test t 30 min**	**β-carotene bleaching test t 60 min**	**FRAP test**
Total phenols content	0.21	0.55			
Total flavonoids content			0.72	0.62	0.67
Total carotenoids content	0.57	0.45			
Ascorbic acid			0.48	0.42	0.69
Caffeic acid			0.51	0.49	0.81
Protocatechuic acid			0.49	0.44	0.80
p-Coumaric acid			0.75	0.82	0.73
Chlorogenic acid	0.54	0.15			
Vanillic acid	0.55	0.41			
Eriocitrin			0.78	0.79	0.47
Gallic acid	0.50	0.09	0.08	0.13	0.26
Apigenin			0.31	0.27	0.33
Didymin		0.13	0.20	0.13	0.31
Quercetin			0.39	0.46	0.42
Hesperidin					0.34
Neohesperidin	0.21		0.18	0.32	0.20
Neoeritrocin	0.18	0.24	0.15	0.04	0.26
Naringin			0.87	0.86	0.73
Narirutin	0.87			0.86	0.73
Poncirin			0.66	0.60	0.67
Sinensetin					0.56
Tangeritin			0.62	0.64	0.79

Table S5. Enriched juice quality parameters

Parameters	°Brix	pH	Acidity (g/100mL)	Croma value (C*)	Total phenols content[1]	Total flavonoids content[2]	Total carotenoids content[3]
JF	10.24 ± 1.03^{a}	3.72 ± 0.07^{a}	0.70 ± 0.02^{bcd}	34.04 ± 2.02^{b}	31.12 ± 1.13^{i}	18.23 ± 0.76^{o}	54.65 ± 1.44^{o}
JFA	10.23 ± 1.14^{a}	3.62 ± 0.04^{f}	0.71 ± 0.03^{a}	33.71 ± 2.00^{f}	41.01 ± 1.04^{a}	28.44 ± 1.04^{a}	61.56 ± 1.76^{e}
JFB	10.22 ± 1.31^{a}	3.64 ± 0.06^{f}	0.71 ± 0.02^{ab}	33.82 ± 2.03^{ef}	40.26 ± 1.16^{b}	26.11 ± 1.15^{c}	59.78 ± 1.43^{f}
JFC	10.22 ± 1.02^{a}	3.68 ± 0.05^{c}	0.70 ± 0.02^{abc}	33.94 ± 2.02^{cde}	38.55 ± 1.12^{cd}	24.01 ± 1.12^{f}	57.89 ± 1.17^{i}
JFD	10.22 ± 1.04^{a}	3.7 ± 0.06^{ab}	0.71 ± 0.04^{abcd}	34.02 ± 2.10^{b}	34.34 ± 1.12^{g}	20.55 ± 1.01^{i}	56.76 ± 1.26^{m}
JFE	10.25 ± 1.12^{a}	3.65 ± 0.07^{ed}	0.71 ± 0.02^{abcd}	33.94 ± 2.09^{cde}	39.10 ± 1.07^{c}	24.34 ± 1.12^{e}	62.45 ± 2.23^{b}
JFF	10.24 ± 1.03^{a}	3.67 ± 0.05^{cd}	0.69 ± 0.03^{d}	34.01 ± 2.11^{bc}	38.01 ± 1.43^{d}	22.32 ± 1.10^{g}	61.98 ± 1.99^{d}
JFG	10.23 ± 1.13^{a}	3.68 ± 0.07^{bc}	0.69 ± 0.03^{cd}	34.04 ± 2.02^{b}	37.43 ± 1.12^{e}	20.43 ± 1.28^{l}	58.89 ± 1.11^{g}
JFH	10.22 ± 1.20^{a}	3.71 ± 0.03^{a}	0.71 ± 0.04^{abcd}	34.08 ± 2.07^{bc}	33.21 ± 1.25^{h}	19.78 ± 0.87^{m}	56.55 ± 1.56^{n}
JFI	10.24 ± 1.03^{a}	3.68 ± 0.07^{cd}	0.69 ± 0.03^{d}	33.87 ± 2.04^{e}	40.34 ± 1.11^{b}	26.53 ± 1.12^{b}	62.15 ± 1.43^{c}
JFL	10.23 ± 1.03^{a}	3.69 ± 0.05^{bc}	0.69 ± 0.03^{bcd}	33.91 ± 2.06^{de}	39.76 ± 1.12^{b}	25.22 ± 1.15^{d}	61.89 ± 1.32^{a}
JFM	10.22 ± 1.04^{a}	3.70 ± 0.08^{ab}	0.70 ± 0.04^{abcd}	34.97 ± 2.12^{a}	35.31 ± 1.21^{f}	21.32 ± 1.04^{h}	58.23 ± 1.65^{h}
JFN	10.22 ± 1.02^{a}	3.70 ± 0.08^{ab}	0.71 ± 0.04^{bcd}	34.01 ± 2.07^{bc}	33.46 ± 1.12^{h}	19.34 ± 0.45^{n}	57.34 ± 1.12^{l}

[1]mg equivalents of chlorogenic acid/100 mL; [2]mg equivalents of quercetin/100 mL; [3]mg equivalents of β-carotene/100 mL. Data represent means ± SD (standard deviation) (n = 3). Differences were evaluated by one-way analysis of variance (ANOVA) test completed with a multicomparison Tukey's test. ** $p < 0.05$. Means in the same column with different small letters differ significantly ($p < 0.05$).

Table S6. Pasteurized enriched juice quality parameters

Samples	°Brix	pH	Acidity (g/100mL)	Croma value (C*)
JPF	10.11 ± 1.52^{cb}	3.70 ± 0.53^{a}	0.75 ± 0.07^{ab}	30.90 ± 1.91^{a}
JPFA	10.09 ± 1.51^{c}	3.71 ± 0.53^{a}	0.76 ± 0.08^{a}	29.04 ± 1.88^{b}
JPFE	10.13 ± 1.53^{a}	3.69 ± 0.51^{a}	0.75 ± 0.06^{ab}	29.51 ± 1.87^{b}
JPFI	10.11 ± 1.52^{ab}	3.7 ± 0.52^{a}	0.74 ± 0.07^{b}	29.16 ± 1.86^{b}

Data represent means ± SD (standard deviation) (n = 3). Differences were evaluated by one-way analysis of variance (ANOVA) test completed with a multicomparison Tukey's test. **p< 0.05. Means in the same column with different small letters differ significantly (p< 0.05).

Table S7. Results of sensory analysis of clementine pasteurized enriched juice

Samples	Appearance	Colour	Odour	Aroma	Sweetness	Acidity	Astringency	Mouthfeel
JF	7.59^{c}	7.94^{b}	7.87^{a}	8.10^{a}	8.01^{a}	7.76^{a}	7.93^{a}	7.89^{a}
JFA	7.47^{c}	7.93^{b}	7.81^{a}	7.81^{c}	7.87^{c}	7.71^{c}	7.84^{b}	7.73^{c}
JFE	8.11^{a}	8.25^{a}	8.07^{a}	7.86^{c}	7.98^{b}	7.73^{b}	7.86^{b}	7.86^{b}
JFI	7.61^{b}	7.96^{b}	7.86^{a}	7.91^{b}	8.00^{a}	7.74^{b}	7.85^{b}	7.85^{b}

Data represent means ± SD (standard deviation) (n = 3). Differences were evaluated by one-way analysis of variance (ANOVA) test completed with a multicomparison Tukey's test. **$p < 0.05$. Means in the same column with different small letters differ significantly ($p < 0.05$).
